# Optimization-Based Online Initialization and Calibration of Monocular Visual-Inertial Odometry Considering Spatial-Temporal Constraints

**DOI:** 10.3390/s21082673

**Published:** 2021-04-10

**Authors:** Weibo Huang, Weiwei Wan, Hong Liu

**Affiliations:** 1 Key Laboratory of Machine Perception, Peking University Shenzhen Graduate School, Shenzhen 518055, China; weibohuang@pku.edu.cn; 2 School of Engineering Science, Osaka University, Osaka 5608531, Japan

**Keywords:** online initialization, spatial-temporal calibration, incremental estimation, monocular visual-inertial odometry

## Abstract

The online system state initialization and simultaneous spatial-temporal calibration are critical for monocular Visual-Inertial Odometry (VIO) since these parameters are either not well provided or even unknown. Although impressive performance has been achieved, most of the existing methods are designed for filter-based VIOs. For the optimization-based VIOs, there is not much online spatial-temporal calibration method in the literature. In this paper, we propose an optimization-based online initialization and spatial-temporal calibration method for VIO. The method does not need any prior knowledge about spatial and temporal configurations. It estimates the initial states of metric-scale, velocity, gravity, Inertial Measurement Unit (IMU) biases, and calibrates the coordinate transformation and time offsets between the camera and IMU sensors. The work routine of the method is as follows. First, it uses a time offset model and two short-term motion interpolation algorithms to align and interpolate the camera and IMU measurement data. Then, the aligned and interpolated results are sent to an incremental estimator to estimate the initial states and the spatial–temporal parameters. After that, a bundle adjustment is additionally included to improve the accuracy of the estimated results. Experiments using both synthetic and public datasets are performed to examine the performance of the proposed method. The results show that both the initial states and the spatial-temporal parameters can be well estimated. The method outperforms other contemporary methods used for comparison.

## 1. Introduction

**Figure 1:**
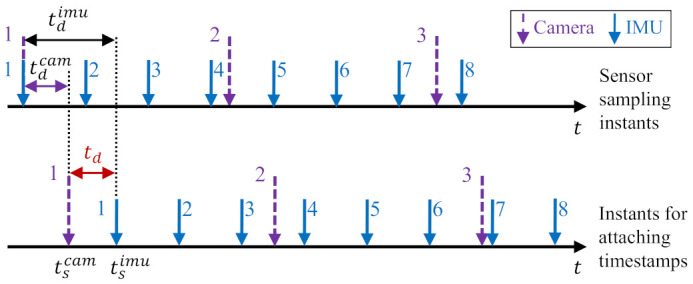
An example of temporal misalignment in the sensor data. The upper plot represents the sensor sampling instants. The lower plot shows instants for attaching timestamps. The timestamped data are essentially the sensor measurements obtained by users. The meanings of the notations are as follows. $t_{d}^{imu}$ and $t_{d}^{cam}$ are, respectively, the latency of Inertial Measurement Unit (IMU) and camera. $t_{d}=t_{d}^{imu}-t_{d}^{cam}$ is the time offset between the stamped IMU and camera data. These two timestamped data can be aligned by shifting the camera data with $t_{d}$ offset or shifting the IMU data with $-t_{d}$ offset.

Monocular Visual-Inertial Odometry (VIO) is an important topic in the robotics and the computer vision community. Its goal is to estimate the incremental motion and reconstruct scene structure by fusing measurements collected from a camera and an Inertial Measurement Unit (IMU). Previously, VIO has been successfully used in applications like unmanned aerial robots [1,2], autonomous or semi-autonomous driving [3,4], 3D reconstruction [5,6], and augmented reality [7,8]. The performance of monocular VIOs heavily relies on the accuracy of the initial states (including metric-scale, velocity, gravity, gyroscope bias, and accelerometer bias) and the spatial-temporal parameters between the camera and IMU. Thus, developing convenient and efficient methods to exactly acquire the values of these parameters is highly demanded. First, an online initialization process is needed to estimate the initial states for bootstrapping the fusion process, since otherwise, the metric-scale of camera measurements is ambiguous, which may cause the optimization sink into a local minimal solution. Second, the spatial-temporal parameters must be both calibrated. In particular, the temporal parameters are used to align different sensor data. The timestamps of the sensor data are taken either from an internal clock in the sensor or from the Operating System (OS) that receives the sensor data. Due to the unsynchronized clocks, transmission delays, sensor response, and OS overhead, latency exists between the actual sampling instant of the sensor data and the timestamp of the captured data; thus, the captured data (i.e., the measurement data) from the camera and IMU are usually misaligned. An example of temporal misalignment in the sensor data is shown in Figure 1. If the spatial-temporal parameters are not considered or incorrectly calibrated, the performance of mapping and navigation would be significantly impaired.

There have been several publications studying the initialization and calibration problems. The early ones [9,10,11,12,13] used offline methods to obtain the spatial-temporal parameters. They required a professional user to carefully move the sensor suite in front of a stationary visual calibration target, which is troublesome in usual deployment and dangerous in cases of emergency. To overcome the disadvantages, several online methods were developed. The online methods assumed that the measurements from a camera and an IMU were well synchronized (e.g., [14,15,16,17,18,19]), or the extrinsic spatial parameter was known in advance (e.g., [20,21,22]), or both conditions were satisfied (e.g., [23,24,25,26,27,28]). In the case where both the measurements from different sensors are asynchronous and the extrinsic spatial parameter between different sensors is unknown, most of the existing methods [29,30,31,32,33,34] are designed for filter-based VIOs since they are usually built on the Multi-State Constraint Kalman Filter (MSCKF [35]) framework. They perform the state propagation/prediction by integrating IMU measurements and perform the state update/correction by using visual measurements. However, for the optimization-based VIOs, which use Bundle Adjustment (BA) to minimize the IMU preintegration errors and feature/photometric reprojection errors and thus have higher accuracy than filter-based ones, there is not much online simultaneous spatial-temporal calibration work in the literature.

Motivated by the situation, we in this paper propose an online initialization and calibration method for optimization-based VIO considering spatial-temporal constraints. The method simultaneously estimates the initial states and calibrates the spatial-temporal parameters during the system bootstrapping. It does not need professional knowledge and tedious offline preparations, thus enabling us to build “power-on-and-go” robots that can operate autonomously and instantaneously. The proposed method is carried out sequentially in three steps. First, our method introduces a time offset model and proposes two short-term sensor motion interpolation algorithms for aligning the asynchronous camera and IMU measurements, based on an assumption that the sensor suite moves in constant angular and linear velocities between consecutive frames with short-term intervals. The two sensor motion interpolation algorithms, i.e., the camera motion interpolation and the IMU motion interpolation, respectively, interpolate the camera pose and IMU pose at arbitrary intermediate times inside each short-term interval. By further considering the unknown metric-scale of camera pose in the camera motion interpolation algorithm, the transformation relationship between the camera and IMU at any timestamp is formulated as a function of the temporal parameter. Second, an incremental estimator considering the temporal misalignment between different sensor data is introduced to estimate the initial states and the spatial-temporal parameters. The incremental estimator is performed repeatedly each time a new keyframe is detected. It first estimates the extrinsic rotation, time offset, and gyroscope bias by minimizing the rotation difference between the camera and IMU. Then, it estimates metric-scale, gravity, and spatial translation by ignoring accelerometer bias. Finally, it refines these values and further estimates the accelerometer bias by taking the gravitational magnitude into account. Third, a bundle adjustment is used to improve the accuracy of the initialization and fuse the visual and inertial measurements. By minimizing both the IMU preintegration error and the feature reprojection error, it can globally or locally optimize the IMU states (including position, rotation, velocity, and biases), the spatial-temporal parameters, and the reconstructed map.

The main contributions of this article are in algorithmic implements:

- We propose an online method for bootstrapping the optimization-based monocular VIO system, which can simultaneously estimate the initial states and calibrate the spatial and temporal parameters between the camera and IMU sensors.
- The time offset is modeled, and two short-term motion interpolation algorithms considering both the temporal parameter and the unknown metric-scale are proposed to interpolate the camera and IMU pose at an arbitrary intermediate time, which enable us to align the camera poses and IMU poses.
- A three-step incremental estimator is introduced to estimate the initial states and the spatial-temporal parameters using the aligned poses.
- A tightly-coupled nonlinear optimization algorithm is additionally included to improve the accuracy of the estimated results.

**Figure 2:**
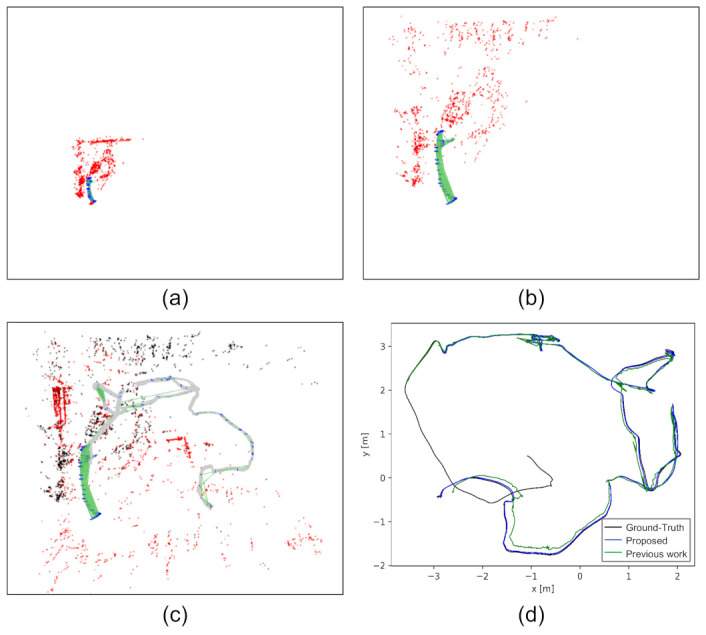
An example of the estimated maps before and after applying the proposed method. The data are from the V2_01 sequence of the EuRoC dataset [37] with a time offset of 45 ms. (**a**) The map estimated by monocular VO. It is subjected to ambiguous scales. (**b**) The map after initialization. The metric-scale was recovered. (**c**) The map constructed by the proposed method on the whole sequence. (**d**) A top-view of the estimated trajectory (blue curve) compared with the ground-truth (black curve) and our previous work [36] (green curve, which calibrates the extrinsic spatial parameter only). **Best viewed in color**.

In the experimental section, quantitative analysis and comparison using synthetic sequences, public datasets, and real visual-inertial sensors are performed to examine the proposed method. The results show the efficacy and efficiency in estimating both the initial states and extrinsic spatial-temporal parameters. For intuitive understanding, an example of the estimated maps before and after applying the proposed method is shown in Figure 2. The map estimated by a pure monocular visual odometry (VO) front-end during initialization is shown in Figure 2a. It is subject to a problem of ambiguous scale. The metric-scale can be recovered by applying the proposed incremental estimator, and a real-sized map is found, as shown in Figure 2b. After initialization, the subsequent measurements are processed by the visual-inertial bundle adjustment. The result is a stitched map shown in Figure 2c. The top-view of the estimated trajectory compared with the ground-truth and our previous work [36] that considered spatial constraint only is shown in Figure 2d, which indicates that the trajectory estimated by simultaneously considering spatial-temporal constraints is more accurate.

The remaining part of this paper is organized as follows: Section 2 reviews the related work. Section 3 presents the time offset model, the short-term sensor motion interpolation algorithms, and the transformation equations developed based on the model and interpolations. Section 4 introduces the preliminary knowledge about the IMU measurement model and the IMU preintegration and presents the incremental estimation of the initial states and the extrinsic spatial-temporal parameters. Section 5 presents the bundle adjustment. Experiments and analyses are performed in Section 6. Conclusions and future work are described in Section 7.

## 2. Related Work

According to the working routine, previous VIO calibration methods can be divided into offline and online methods. Offline methods have a long history. For example, Mirzaei et al. [38] performed an observability analysis for rigid transformation between the camera and IMU. They showed that under certain motion, the rigid transformation is not fully observable. Furgale et al. [9,10] developed an open-source camera/IMU calibration toolbox named *Kalibr* (https://github.com/ethz-asl/kalibr, accessed on 14 February 2021), to perform the temporal and spatial calibration for a visual-inertial sensor in an offline manner. By using a continuous-time basis function representation [39] of the sensor trajectory, Rehder et al. [12,13] extended the toolbox to calibrate both the extrinsic and intrinsic parameters of multiple IMUs.The drawback of offline calibration is the need for additional calibration steps and assistive devices. In addition, a recalibration must be performed every time the configuration of a sensor suite is changed. To avoid inconvenience, researchers switched to online methods.

Online methods estimate the extrinsic parameters at the arrival of each new sensor measurements. It invalidates the need for assistive devices and, at the same time, maintains high accuracy, thereby making the odometry more robust and easier to use. According to the unknown parameters, the online method for monocular VIOs can be divided into online spatial calibration, online temporal calibration, and simultaneous online spatial-temporal calibration.

For online spatial calibration, Kelly et al. [15] proposed a self-calibration method based on the unscented Kalman filter. The method showed that the full observability of spatial parameters required the sensor suite to undergo both the rotation and acceleration on at least two IMU axes. Li et al. [16,17] estimated the spatial parameters along with a sliding window of sensor poses using an extended Kalman filter algorithm. It achieved consistent estimation by ensuring the correct observability properties of its linearized system model. Yang and Shen [18] calibrated the spatial parameters and the initial states (except for IMU biases) with an optimization-based linear estimator. In their extended batch-based monocular VINS (termed VINS-Mono) [40], the IMU biases are included in a sliding window nonlinear estimator. Schneider et al. [41] introduced information-theoretic metrics to assess the information content of trajectory segments, thus allowing them to select the most informative parts from a dataset for extrinsic spatial calibration purposes. Huang et al. [19] proposed an estimator to incrementally solve several linear equations to estimate the spatial parameters between an IMU and two cameras.

For online temporal calibration, Kelly et al. [20] formulated the calibration problem as a registration task. Their method leveraged a variant of iterative closet point algorithm, termed TD-ICP, to gradually match the three-dimensional orientation curves of camera and IMU. Mair et al. [42] presented and compared two approaches, i.e., cross-correlation and phase congruence, to temporally align the camera and the gyroscope, which showed that the cross-correlation based approach was more suitable for short delay. Ling et al. [21] presented a time-varying model for temporal calibration. This approach can handle the rolling-shutter effects and imperfect sensor synchronization in a nonlinear optimization algorithm. Qin et al. [22] extended [40] to include estimating the time offset between the camera and IMU by interpolating the location of image features. The method treated the time offset as a vision factor and calibrated it online along with features and sensor poses in an optimization-based VIO framework. Liu et al. [34] proposed an online temporal calibration method that was integrated into the filter-based VIO framework (S-MSCKF [43]). To ensure the consistency between the IMU measurement constraint and the visual measurement constraint, the authors established a modified feature projection model considering time offset and introduced an interpolation-based algorithm for inferring the 2D observations of features on a virtual image.

Although various sophisticated online approaches for either spatial calibration or temporal calibration have been proposed, drawbacks remain. Online spatial calibration methods [15,16,17,18,19] require the measurements from different sensors to be strictly synchronized, while online temporal calibration methods [20,21,22] assume the relative transformations between different sensors are prerequisites. In cases of low-cost and self-assembled devices, accurate factory calibration and hardware synchronization are not available. Such devices inspire the study of simultaneous online spatial-temporal calibration. For example, Li et al. [29,30] treated the time offset as an additional state variable to be estimated along with IMU pose, velocity, biases, feature positions, and extrinsic spatial parameters. To compute the feature residual, the authors performed the propagation using the IMU measurements up to the estimated time offset. Li’s works also showed that the time offset was locally identifiable, except in a small number of degenerate cases like constant-velocity motion. Zuo et al. [31] developed a Lidar-inertial-camera odometry that could refine the spatial-temporal parameters online along with sensor pose estimation. Yang et al. [32] analyzed the observability of spatial-temporal parameters and showed that they were both observable if the sensor platform underwent fully random motion. The authors also identified four degenerate motions that were harmful to the calibration accuracy. Eckenhoff et al. [33] used interpolation on the SO(3) manifold to represent anytime IMU poses and successfully performed the spatial-temporal calibration for a multi-camera system. The short-term motion interpolation used in our paper is inspired by [33]. We extend it to interpolate the to-scale camera poses and thus can represent anytime camera poses and express the IMU pose by transforming the camera pose at the *true* time. We estimate the initial states and extrinsic parameters using the true time IMU-camera pose relations.

Although impressive performance has been achieved in the above-mentioned simultaneous online spatial-temporal calibration methods, most of them are designed for filter-based VIOs. For optimization-based VIOs, there is not much online spatial-temporal calibration method in the literature. Motivated by this situation, we, in this paper, propose an incremental estimator that simultaneously considers the spatial-temporal constraints and develops optimization-based algorithms. Our method can solve the calibration problem when both the spatial and temporal parameters are unknown. To the best of our knowledge, similar methods are less seen in modern literature. The only publication that has a similar conception is Feng’s method [44], which estimated the extrinsic rotation and time offset by minimizing the quaternion rotation difference between the camera and IMU. The method used a loosely coupled approach described in [45] to recover the initial states and used a nonlinear optimization algorithm to minimize sensor noises. Different from Feng’s method, our focus is the time offset among the sensors. We model the time offset and use the temporal constraints to interpolate the poses of the camera and IMU. The systematic accuracy and robustness are improved by taking the time offset into account. Compared with [22,30,34], our method directly performs the motion interpolation on the camera poses and IMU poses for compensating the time offset. By considering the time offset and the unknown metric-scale factor in the camera motion interpolation algorithm, it benefits us to inherit the incremental estimation routine introduced in our previous work [36]. Owing to the interpolation on camera poses, the proposed incremental estimator can be easily applied to the popular pure visual SLAM/Odometry frameworks (e.g., LSD-SLAM [46], ORB-SLAM [47,48,49], DSO [50], SVO [51,52]) to conduct visual-inertial systems since the input of our estimator is merely the IMU measurements and the keyframe poses. These frameworks can provide accurate up-to-scale camera pose and serve as the VO front-end for our incremental estimator.

## 3. Short-Term Sensor Motion Interpolation

This section introduces the short-term sensor motion interpolation algorithm. First, the time offset model used to align the camera and IMU data are presented. Then, two motion interpolation algorithms are introduced to, respectively, interpolate the camera and IMU poses. After that, the transformation relationship between the camera and IMU is derived by using the interpolated poses.

The meanings of the various symbols used in this section are as follows. ${\left(\cdot \right)}^{w}$ denotes the states expressed in the world reference frame {w}. It coincides with the camera coordinate system of the first keyframe. $T_{c_{i}}^{w}=\left[R_{c_{i}}^{w}|s\cdot p_{c_{i}}^{w}\right]$ is the pose of the camera frame $\left\{c_{i}\right\}$ expressed in {w} at time $t_{i}$, where $R_{c_{i}}^{w}\in \mathrm{SO}\left(3\right)$ and $p_{c_{i}}^{w}\in R^{3}$ are, respectively, the camera rotation and position. *s* is the metric-scale used to present the scale ambiguity in the camera position. $T_{b_{i}}^{w}=\left[R_{b_{i}}^{w}|p_{b_{i}}^{w}\right]$ is the pose of the IMU body frame $\left\{b_{i}\right\}$ expressed in {w} at time $t_{i}$. $T_{c}^{b}=\left[R_{c}^{b}|p_{c}^{b}\right]$ is the relative transformation between the camera and IMU, i.e., the extrinsic spatial parameter that should be calibrated. For convenience, the inverse representations, e.g., $T_{b}^{c}=\left[R_{b}^{c}|p_{b}^{c}\right]=\left[{R_{c}^{b}}^{T}|-{R_{c}^{b}}^{T}\cdot p_{c}^{b}\right]$, are also used in some equations. The time offset between the camera and IMU measurements is denoted as $t_{d}$. The relative transformation $T_{c}^{b}$ and time offset $t_{d}$ are fixed but unknown.

### 3.1. Time Offset Model

As previously shown in Figure 1, the camera and IMU in a monocular VIO sensor suite, respectively, provide discrete samplings in a constant frequency. Due to the unsynchronized clocks, transmission delays, sensor response, and operating system overhead, there exists a latency that makes the measured (i.e., timestamped) data misalign with the sampling instants. Consider the camera and the IMU measurements sampled at the same instant *t*, their timestamps $t_{s}^{cam}$ and $t_{s}^{imu}$ can be modeled as:

(1) $$t_{s}^{cam}=t+t_{d}^{cam},\;\;\;\;t_{s}^{imu}=t+t_{d}^{imu},$$

where $t_{d}^{cam}$ and $t_{d}^{imu}$ are, respectively, the latencies of camera and IMU. The time offset $t_{d}$ thus equals to:

(2) $$t_{d}≐t_{d}^{imu}-t_{d}^{cam}=t_{s}^{imu}-t_{s}^{cam}.$$

Equations (1) and (2) indicate that the camera and IMU data can be aligned by shifting the camera data with $t_{d}$ offset or shifting the IMU data with $-t_{d}$ offset, namely:

(3) $$\begin{array}{c} T_{b_{i}}^{w}=T_{c_{i,t_{d}}}^{w}\cdot T_{b}^{c}, \end{array}$$

(4) $$\begin{array}{c} T_{c_{i}}^{w}=T_{b_{i,-t_{d}}}^{w}\cdot T_{c}^{b}. \end{array}$$

**Figure 3:**
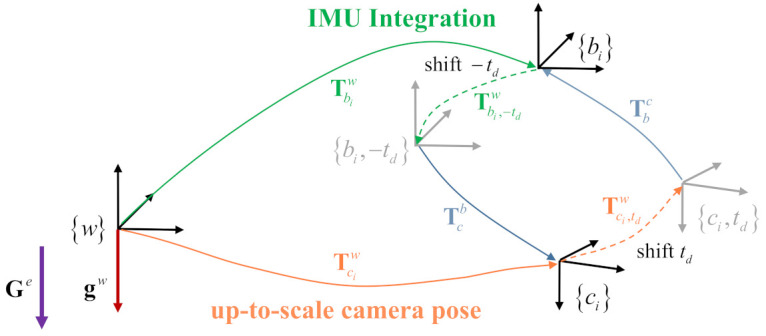
Transformation relationships between the camera and IMU coordinate frames when considering the time offset. The camera and IMU poses can be aligned by shifting the camera pose with $t_{d}$ offset or shifting the IMU pose with $-t_{d}$ offset. The world reference frame {w} coincides with the first keyframe’s coordinate system of the monocular visual odometry front-end. $G^{e}$ is the gravity expressed in the earth inertial reference frame {e}.

Here, $T_{c_{i,t_{d}}}^{w}=\left[R_{c_{i},t_{d}}^{w}|s\cdot p_{c_{i},t_{d}}^{w}\right]$ is the camera pose at time $t_{i}+t_{d}$. $T_{b_{i,-t_{d}}}^{w}=\left[R_{b_{i},-t_{d}}^{w}|p_{b_{i},-t_{d}}^{w}\right]$ is the IMU pose at time $t_{i}-t_{d}$. Equation (3) indicates that an IMU pose at time $t_{i}$ is corresponded to the camera pose at time $t_{i}+t_{d}$. Equation (4) indicates that a camera pose at time $t_{i}$ corresponds to the IMU pose at time $t_{i}-t_{d}$. The transformation relationships between the camera and IMU coordinate frames when considering the time offset are illustrated in Figure 3.

### 3.2. Sensor Motion Interpolation

#### 3.2.1. Camera Motion Interpolation

In this work, the camera is assumed to move in constant angular and linear velocities in a short period; therefore, the camera pose at an arbitrary time can be interpolated using its nearest camera poses. Consider two up-to-scale camera poses $T_{c_{i}}^{w}$ and $T_{c_{j}}^{w}$ estimated at timestamp $t_{i}$ and $t_{j}$; the camera angular velocity $\omega_{c_{i}}$ and linear velocity ${\tilde{v}}_{c_{i}}$ at $t_{i}$ are approximately

(5) $$\begin{array}{cc} \omega_{c_{i}} & \approx \mathrm{Log}\left({R_{c_{i}}^{w}}^{T}R_{c_{j}}^{w}\right)/\left(t_{j}-t_{i}\right),\\ {\tilde{v}}_{c_{i}} & \approx \left(p_{c_{j}}^{w}-p_{c_{i}}^{w}\right)/\left(t_{j}-t_{i}\right), \end{array}$$

where Log(·) is the “vectorized” version of *logarithm map* that transforms a rotation matrix R≠I to a vector ϕ, with $\phi =\mathrm{Log}\left(R\right)=\ln{\left(R\right)}^{\vee }$ and ${\left(\cdot \right)}^{\vee }$ is the *vee* operator that maps a skew-symmetric matrix in $R^{3\times 3}$ to a vector in $R^{3}$. Its reverse process is called a *hat* operator ${\left(\cdot \right)}^{\wedge }$ that maps a vector in $R^{3}$ to a skew-symmetric matrix. A property of skew-symmetric matrices that will be used is as follows: given two vectors $a,b\in R^{3}$, the cross-product can be expressed as $a\times b=a^{\wedge }\cdot b=-b^{\wedge }\cdot a$. Note that the velocity term ${\tilde{v}}_{c_{i}}$ in the equation is computed using the up-to-scale camera positions. The metric-scale factor is taken into account in the derivation of spatial relationships between camera and IMU (see Equation (9)).

Using Equation (5), the camera rotation and position at time $t_{i}+t_{d}$ can be interpolated as follows:

(6) $$\begin{array}{cc} R_{c_{i},t_{d}}^{w} & \approx R_{c_{i}}^{w}\mathrm{Exp}\left(\omega_{c_{i}}t_{d}\right),\\ p_{c_{i},t_{d}}^{w} & \approx p_{c_{i}}^{w}+{\tilde{v}}_{c_{i}}t_{d}. \end{array}$$

#### 3.2.2. IMU Motion Interpolation

Similarly, assuming the IMU moves in constant angular and linear velocities in a short period, the rotation and position at time $t_{i}-t_{d}$ can be interpolated as:

(7) $$\begin{array}{cc} R_{b_{i},-t_{d}}^{w} & \approx R_{b_{i}}^{w}\mathrm{Exp}\left(-{\bar{\omega }}_{b_{i}}t_{d}\right),\\ p_{b_{i},-t_{d}}^{w} & \approx p_{b_{i}}^{w}-v_{b_{i}}^{w}t_{d}, \end{array}$$

where ${\bar{\omega }}_{b_{i}}$ and $v_{b_{i}}^{w}$ are, respectively, the angular and linear velocities of the IMU at $t_{i}$.

### 3.3. Spatial Relationships between Camera and IMU

By considering the time offset $t_{d}$ and the metric-scale *s*, the rotation and position of the IMU at timestamp $t_{i}$ can be derived from a camera pose according to Equation (3) as follows:

(8) $$\begin{array}{cc} R_{b_{i}}^{w} & =R_{c_{i},t_{d}}^{w}R_{b}^{c}\approx R_{c_{i}}^{w}\mathrm{Exp}\left(\omega_{c_{i}}t_{d}\right)R_{b}^{c}, \end{array}$$

(9) $$\begin{array}{c} \begin{array}{cc} p_{b_{i}}^{w} & =R_{c_{i},t_{d}}^{w}p_{b}^{c}+s\cdot p_{c_{i},t_{d}}^{w}\\ \; & \approx R_{c_{i}}^{w}\mathrm{Exp}\left(\omega_{c_{i}}t_{d}\right)p_{b}^{c}+s\cdot \left(p_{c_{i}}^{w}+{\tilde{v}}_{c_{i}}t_{d}\right). \end{array} \end{array}$$

Similarly, the camera pose at timestamp $t_{i}$ can be derived from an IMU pose according to Equation (4), as follows:

(10) $$\begin{array}{cc} R_{c_{i}}^{w} & =R_{b_{i},-t_{d}}^{w}R_{c}^{b}\approx R_{b_{i}}^{w}\mathrm{Exp}\left(-{\bar{\omega }}_{b_{i}}t_{d}\right)R_{c}^{b}, \end{array}$$

(11) $$\begin{array}{c} \begin{array}{cc} p_{c_{i}}^{w} & =R_{b_{i},-t_{d}}^{w}p_{c}^{b}+p_{b_{i},-t_{d}}^{w}\\ \; & \approx R_{b_{i}}^{w}\mathrm{Exp}\left(-{\bar{\omega }}_{b_{i}}t_{d}\right)p_{c}^{b}+p_{b_{i}}^{w}-v_{b_{i}}^{w}t_{d}. \end{array} \end{array}$$

In the following sections, Equations (8) and (9) are leveraged in the incremental estimator. Equations (10) and (11) are used for the bundle adjustment. Note that since the metric-scale can be recovered by performing the incremental estimator and the camera position term $p_{c_{i}}^{w}$ in (11) is transformed from the IMU pose, the metric-scale term is removed.

## 4. Online Initialization and Extrinsic Spatial-Temporal Calibration

This section details how to combine the motion interpolation algorithms introduced in Section 3 and the estimator introduced in our previous work [36] to estimate the initial states and the spatial-temporal parameters.

For the reader’s convenience, we first briefly introduce the IMU preintegration theory in Section 4.1. Then, the incremental estimator for online initialization is derived in Section 4.2. The incremental update of time offset, velocity estimation, termination condition, and implementation note are discussed in Section 4.3.

### 4.1. IMU Preintegration

The idea of preintegrated IMU measurements was first proposed by Lupton et al. [53]. Its essence is to combine many inertial measurements between two keyframes into a single relative motion constraint. Lupton et al. used Euler angles to represent the IMU rotation. The representation was later extended by Forster et al. [54] using a manifold in SO(3) to avoid singularity. In this paper, we follow the IMU measurement model and the kinematic model of the system motion given in [54]. In the following, we denote the IMU body frame as {b} and the world reference frame as {w}, and the gravitational acceleration in {w} as $g^{w}$. The gyroscope outputs $\omega_{b}$ and accelerometer outputs $a_{b}$ are, respectively, subject to white sensor noises $\eta_{g}$ and $\eta_{a}$ (normally assumed as Gaussian noise), and slow time-varying gyroscope bias $b_{g}$ and accelerometer bias $b_{a}$.

Given a series of IMU measurements from time *i* to time *j* (j>i) and ignoring the sensor noises, the changes of IMU rotation $R_{b}^{w}$, velocity $v_{b}^{w}$, and position $p_{b}^{w}$ between the two time instants can be computed by:

(12) $$\begin{array}{cc} R_{b_{j}}^{w} & =R_{b_{i}}^{w}\Delta {\bar{R}}_{ij}\mathrm{Exp}\left(J_{\Delta {\bar{R}}_{ij}}^{g}\delta b_{g_{i}}\right),\\ v_{b_{j}}^{w} & =v_{b_{i}}^{w}\;+\;g^{w}\Delta t_{ij}\;+\;R_{b_{i}}^{w}\;\left(\;\Delta {\bar{v}}_{ij}\;+J_{\Delta {\bar{v}}_{ij}}^{g}\delta b_{g_{i}}\;+J_{\Delta {\bar{v}}_{ij}}^{a}\delta b_{a_{i}}\;\right)\;,\\ p_{b_{j}}^{w} & =p_{b_{i}}^{w}\;+\;v_{b_{i}}^{w}\Delta t_{ij}\;+\;\frac{1}{2}g^{w}\Delta t_{ij}^{2}\\ \; & \;\;\;+R_{b_{i}}^{w}\left(\Delta {\bar{p}}_{ij}\;+\;J_{\Delta {\bar{p}}_{ij}}^{g}\delta b_{g_{i}}\;+\;J_{\Delta {\bar{p}}_{ij}}^{a}\delta b_{a_{i}}\right), \end{array}$$

where Δt is the IMU sampling interval, and $\Delta t_{ij}≐\sum_{k=i}^{j-1}\Delta t$. Exp(·) is the “vectorized” version of *exponential map* that transforms a vector $\phi \in R^{3}$ to a rotation matrix R∈SO(3), with $R=\mathrm{Exp}\left(\phi \right)=\exp\left(\phi^{\wedge }\right)$. In the equation, the Jacobians $J_{\left(\cdot \right)}^{g}$ and $J_{\left(\cdot \right)}^{a}$ indicate how the measurements change with respect to the bias estimation. $\Delta {\bar{R}}_{ij}$, $\Delta {\bar{v}}_{ij}$, and $\Delta {\bar{p}}_{ij}$ are the preintegrated terms during the time period (i,j). They can be computed directly using the IMU measurements as follows:

(13) $$\begin{array}{cc} \Delta {\bar{R}}_{ij} & =\prod_{k=i}^{j-1}\mathrm{Exp}\left(\left(\omega_{b_{k}}-{\bar{b}}_{g_{i}}\right)\Delta t\right),\\ \Delta {\bar{v}}_{ij} & =\sum_{k=i}^{j-1}\Delta {\bar{R}}_{ik}\left(a_{b_{k}}-{\bar{b}}_{a_{i}}\right)\Delta t,\\ \Delta {\bar{p}}_{ij} & =\sum_{k=i}^{j-1}\left(\Delta {\bar{v}}_{ik}\Delta t+\frac{1}{2}\Delta {\bar{R}}_{ik}\left(a_{b_{k}}-{\bar{b}}_{a_{i}}\right)\Delta t^{2}\right). \end{array}$$

Here, the biases are assumed to remain the same during the time period (i,j). They can be represented as $b_{g_{i+1}}=\ldots =b_{g_{j}}\approx {\bar{b}}_{g_{i}}+\delta b_{g_{i}}$, and $b_{a_{i+1}}=\ldots =b_{a_{j}}\approx {\bar{b}}_{a_{i}}+\delta b_{a_{i}}$. ${\bar{b}}_{g_{i}}$ and ${\bar{b}}_{a_{i}}$ are the biases at time *i*. $\delta b_{g_{i}}$ and $\delta b_{a_{i}}$ are compensation terms used to compensate for the time-varying parts. In the following, the preintegrated terms will be used for incremental estimation and constructing the IMU preintegration error term of nonlinear functions.

### 4.2. Incremental Estimation

In the incremental estimator, both the spatial and temporal parameters between the camera and IMU, as well as the initial states of metric-scale, gravity, and IMU biases, are simultaneously estimated. The basic idea is to solve the equality constraints between the up-to-scale camera poses with the integrated IMU poses. First, we run the sensor suite for a few seconds with at least two-axis rotation plus some random translation. Several keyframes are collected in the process. After that, we perform the incremental estimator repeatedly across consecutive keyframes until the values to be estimated converge.

The incremental estimator is indeed developed from the three-step process proposed in our previous work [36]. In [36], the extrinsic spatial parameters and initial values were online-estimated under the assumption that the camera and IMU measurements were well aligned. Therefore, in the first step, it used an iterative strategy to individually estimate the gyroscope bias and the extrinsic rotation matrix. In each iteration, the last estimated extrinsic rotation matrix was utilized to estimate a gyroscope bias. Then the preintegrated rotations were rectified by applying the estimated gyroscope bias. After that, a linear over-determined equation was developed to calculate a new extrinsic rotation matrix. The iterative estimation was performed until the extrinsic parameters converged. In the other two steps, the up-to-scale camera poses were directly used to conduct the position relationship. Unlike the previous work, the measurement misalignment case is taken into account in the current work. In comparison, the incorporation of time offset is not simple addition. It requires changes in all aspects, which makes the steps very different from our published work. IN particular, when asynchrony appears between sensors, it is difficult to distinguish the temporal parameter and the extrinsic rotation matrix, and one cannot individually estimate the gyroscope bias, the temporal parameter, and the extrinsic rotation matrix through an iterative manner. To overcome the difficulty, the camera motion interpolation algorithm is utilized to compensate for the time offset. In the first step of the incremental estimator, the interpolated camera rotations (i.e., the Equation (8)) are adopted; thus the temporal parameter, the extrinsic rotation matrix, and the gyroscope bias can be estimated together by solving an optimization function that minimizes the relative rotation difference between the IMU rotation and the interpolated camera rotation. In the other two steps, we further apply the interpolated camera positions considering the unknown metric-scale factor (i.e., the Equation (9)) to calibrate the extrinsic translation parameters and other initial states from coarse to fine.

#### 4.2.1. Estimating Gyroscope Bias, and Calibrating Extrinsic Rotation and Time Offset

In the incremental estimation, the gyroscope bias is assumed to have a constant value as it changes slowly over time. The rotation relationships of two consecutive keyframes at timestamp *i* and i+1 can be described as:

(14) $$R_{b_{i}}^{w}=R_{c_{i},t_{d}}^{w}R_{b}^{c},\;\;\;R_{b_{i+1}}^{w}=R_{c_{i+1},t_{d}}^{w}R_{b}^{c},$$

where $R_{b_{i}}^{w}$ and $R_{b_{i+1}}^{w}$ are the IMU rotations that are derived by transforming the interpolated camera rotations. By substituting the first equation of (12) into (14) and using the notation i+1 to replace the notation *j*, the difference between the preintegrated rotation and the transformed value is:

(15) $$\begin{array}{cc} e_{rot_{i,i+1}} & =\mathrm{Log}\left({\left(\Delta {\bar{R}}_{i,i+1}\mathrm{Exp}\left(J_{\Delta {\bar{R}}_{i,i+1}}^{g}\delta b_{g}\right)\right)}^{T}\;R_{c}^{b}\right.\\ \; & \left.\cdot \mathrm{Exp}\left(-\omega_{c_{i}}t_{d}\right)R_{w}^{c_{i}}R_{c_{i+1}}^{w}\mathrm{Exp}\left(\omega_{c_{i+1}}t_{d}\right)R_{b}^{c}\right), \end{array}$$

where $R_{b}^{c}$ and $R_{w}^{c_{i}}$ are, respectively, the inverse of $R_{c}^{b}$ and $R_{c_{i}}^{w}$.

With *N* collected keyframes, the gyroscope bias compensation $\delta b_{g}^{*}$, extrinsic rotation ${R_{c}^{b}}^{*}$ and time offset $t_{d}^{*}$ can be estimated by minimizing the rotation difference for all keyframes as follows:

(16) $$\delta b_{g}^{*},{R_{b}^{c}}^{*},t_{d}^{*}=\underset{\delta b_{g},R_{b}^{c},t_{d}}{\arg\min}\sum_{i=1}^{N-1}{∥e_{rot_{i,i+1}}∥}_{\Sigma_{\Delta \;R}}^{2},$$

where $\Sigma_{\Delta \;R}$ is the information matrices associated with the preintegrated rotation. The Equation (16) is a nonlinear least-squares problem on Lie algebra. It can be solved by using the iterative methods, like the Gauss–Newton method or the Levenberg–Marquardt method [55]. In the first estimation, since we do not have any prior knowledge about the time offset and extrinsic rotation, the initial seeds for $t_{d}$ and $\delta b_{g}$ are set to zero, and $R_{b}^{c}$ is set to an identity matrix. After that, the results obtained in the last estimation are used as initial seeds. Note that when Equation (16) is solved, the preintegrated terms described in (13) are re-computed by using the new estimated gyroscope bias, which facilitates the following steps. The Jacobians of $e_{rot_{i,i+1}}$ needed by the iterative method are derived in Appendix A.4.

#### 4.2.2. Approximating Scale, Gravity, and Extrinsic Translation

Once the extrinsic rotation ${R_{c}^{b}}^{*}$ and time offset $t_{d}^{*}$ have been found by Equation (16), the metric-scale *s*, gravity vector $g^{w}$, and extrinsic translation $p_{b}^{c}$ can be approximately estimated by combining Equations (8), (9), and (12).

First, by substituting (8) and (9) into the third equation of (12), the position relationship between two consecutive keyframes *i* and i+1 can be obtained:

(17) $$\begin{array}{cc} s\cdot p_{c_{i+1},t_{d}}^{w} & =s\cdot p_{c_{i},t_{d}}^{w}+v_{b_{i}}^{w}\Delta t_{i,i+1}+\frac{1}{2}g^{w}\Delta t_{i,i+1}^{2}\\ \; & \;\;\;\;\;\;\;\;\;\;\;+R_{c_{i},t_{d}}^{w}{R_{b}^{c}}^{*}\Delta {\bar{p}}_{i,i+1}+\left(R_{c_{i},t_{d}}^{w}\;-\;R_{c_{i+1,t_{d}}}^{w}\right)\cdot p_{b}^{c}, \end{array}$$

where ${R_{b}^{c}}^{*}$ is the result of (16). The items $J_{\Delta \bar{p}}^{a}$ and $J_{\Delta \bar{v}}^{a}$ are temporarily set to zero since the accelerometer bias is not considered for approximation. The accelerometer bias will be estimated in the refinement process. Furthermore, by re-computing the preintegration terms after the gyroscope bias estimation and assuming the gyroscope bias is constant, $J_{\Delta \bar{p}}^{g}$ and $J_{\Delta \bar{v}}^{g}$ can be set to zero. Note that there is a velocity term $v_{b_{i}}^{w}$ in the Equation (17). This term is undetermined and will increase the complexity of the incremental estimation if it is reserved. In the following, we introduce a trick to eliminate the velocity term.

Second, by considering three consecutive keyframes, we can obtain two relations in the form of (17). Using these two relations and substituting the second equation of (12), the velocity terms can be eliminated, as follows:

(18) $$\left[\begin{array}{ccc} \lambda (i) & \beta (i) & \varphi (i) \end{array}\right]\left[\begin{array}{c} s\\ g^{w}\\ p_{b}^{c} \end{array}\right]=\gamma \left(i\right).$$

When writing keyframes *i*, i+1, i+2 as 1, 2, 3, λ(i), β(i), φ(i), and γ(i) can be expressed as:

(19) $$\begin{array}{cc} \lambda (i) & =\left(p_{c_{2},t_{d}}^{w}\;\;-p_{c_{1},t_{d}}^{w}\right)\Delta t_{23}-\left(p_{c_{3},t_{d}}^{w}\;\;-p_{c_{2},t_{d}}^{w}\right)\Delta t_{12},\\ \beta (i) & =\frac{1}{2}\left(\Delta t_{12}\Delta t_{23}^{2}+\Delta t_{12}^{2}\Delta t_{23}\right)I_{3\times 3},\\ \varphi (i) & =\left(R_{c_{2},t_{d}}^{w}\;\;-R_{c_{3},t_{d}}^{w}\right)\Delta t_{12}\;-\left(R_{c_{1},t_{d}}^{w}\;\;-R_{c_{2},t_{d}}^{w}\right)\Delta t_{23},\\ \gamma (i) & =R_{c_{1},t_{d}}^{w}{R_{b}^{c}}^{*}\left(\Delta {\bar{p}}_{12}\Delta t_{23}\;-\Delta {\bar{v}}_{12}\Delta t_{12}\Delta t_{23}\right)\\ \; & \;\;\;-R_{c_{2},t_{d}}^{w}{R_{b}^{c}}^{*}\Delta {\bar{p}}_{23}\Delta t_{12}. \end{array}$$

With *N* keyframes, we can obtain N−2 relations in the form of Equation (18). These relations can be stacked into a linear over-determined system equation $B_{3(N-2)\times 7}\cdot x_{7\times 1}=C_{3(N-2)\times 1}$ with weights for outlier handling [36]. This system equation can be solved using Singular Value Decomposition (SVD) to get the approximate metric-scale $s^{*}$, gravity vector ${g^{w}}^{*}$, and extrinsic translation ${p_{b}^{c}}^{*}$. Note that since there are 3(N−2) sub-equations and a seven-dimension unknown vector in the system equation, at least five keyframes are required to solve it.

Since the accelerometer bias is temporally set to zero in carrying out the computations, the estimated results in this step are approximated. They will be refined in the following part by considering the magnitude of gravitational acceleration and taking the accelerometer bias into account.

#### 4.2.3. Estimating Accelerometer Bias, and Refining Scale, Gravity, and Extrinsic Translation

Next, we estimate the accelerometer bias and refine the scale, gravity, and translation. We denote the earth inertial reference frame as {e} and compare it to the world reference frame {w}. Using the already estimated ${g^{w}}^{*}$ in the approximation process, the rotation between {e} and {w} can be obtained as:

(20) $$\begin{array}{cc} R_{e}^{w} & =\mathrm{Exp}(\vec{n}\alpha ),\\ \vec{n} & =\frac{{\tilde{g}}^{e}\times {\tilde{g}}^{w}}{∥{\tilde{g}}^{e}\times {\tilde{g}}^{w}∥},\;\;\alpha =\mathrm{atan}2(∥{\tilde{g}}^{e}\times {\tilde{g}}^{w}∥,\;{\tilde{g}}^{e}\cdot {\tilde{g}}^{w}),\\ {\tilde{g}}^{w} & ={g^{w}}^{*}/∥{g^{w}}^{*}∥,\;\;{\tilde{g}}^{e}=G^{e}/∥G^{e}∥,\;\;G^{e}={\left[0,\;0,\;-G\right]}^{T}, \end{array}$$

where $\vec{n}$ and α are, respectively, the rotation axis and the rotation angle of $R_{e}^{w}$. $G^{e}$ is the gravity vector expressed in {e}. G is the magnitude of the gravitational acceleration (normally G=9.81 m/s${}^{2}$). This rotation can be optimized by appending a perturbation $\delta \theta \in R^{3\times 1}$, as follows:

(21) $$\begin{array}{cc} g^{w} & =R_{e}^{w}\mathrm{Exp}\left(\delta \theta \right)\cdot G^{e}\;\;\approx \;\;R_{e}^{w}\cdot G^{e}-R_{e}^{w}\cdot {G^{e}}^{\wedge }\cdot \delta \theta ,\;\;\; \end{array}$$

where the first-order approximation of exponential map (see Equation (A2) in Appendix A.1) is applied. By substituting (21) into (17) and further considering the accelerometer bias, we have:

(22) $$\begin{array}{cc} s\;\cdot p_{c_{i+1},t_{d}}^{w} & =s\;\cdot p_{c_{i},t_{d}}^{w}\;+v_{b_{i}}^{w}\Delta t_{i,i+1}\;-\frac{1}{2}R_{e}^{w}\;\cdot {G^{e}}^{\wedge }\;\cdot \delta \theta \Delta t_{i,i+1}^{2}\\ \; & \;\;\;+R_{c_{i},t_{d}}^{w}{R_{b}^{c}}^{*}\left(\Delta {\bar{p}}_{i,i+1}\;+J_{\Delta {\bar{p}}_{i,i+1}}^{a}\delta b_{a}\right)\\ \; & \;\;\;+\left(R_{c_{i},t_{d}}^{w}\;-R_{c_{i+1},t_{d}}^{w}\right)\cdot p_{b}^{c}+\frac{1}{2}R_{e}^{w}\;\cdot G^{e}\Delta t_{i,i+1}^{2}. \end{array}$$

Similar to (18), the velocity term can be eliminated by considering three consecutive keyframes and applying the second equation of (12), which results in:

(23) $$\left[\begin{array}{cccc} \lambda (i) & \phi (i) & \zeta (i) & \xi (i) \end{array}\right]\left[\begin{array}{c} s\\ \delta \theta_{xy}\\ \delta b_{a}\\ p_{b}^{c} \end{array}\right]=\psi \left(i\right).$$

Here, λ(i) remains the same as in (19), and ϕ(i), ζ(i), ξ(i), and ψ(i) are computed as follows:

(24) $$\begin{array}{cc} \phi (i) & ={\left[-\frac{1}{2}R_{e}^{w}\;\cdot \;{G^{e}}^{\wedge }\;\cdot \;\left(\Delta t_{12}\Delta t_{23}^{2}+\Delta t_{12}^{2}\Delta t_{23}\right)\right]}_{\left(:,1:2\right)},\\ \zeta (i) & =R_{c_{1},t_{d}}^{w}{R_{b}^{c}}^{*}\left(J_{\Delta {\bar{v}}_{12}}^{a}\Delta t_{12}\Delta t_{23}-J_{\Delta {\bar{p}}_{12}}^{a}\Delta t_{23}\right)\\ \; & \;\;\;+R_{c_{2},t_{d}}^{w}{R_{b}^{c}}^{*}J_{\Delta {\bar{p}}_{23}}^{a}\Delta t_{12},\\ \xi (i) & =\left(R_{c_{2},t_{d}}^{w}-R_{c_{3},t_{d}}^{w}\right)\Delta t_{12}-\left(R_{c_{1},t_{d}}^{w}-R_{c_{2},t_{d}}^{w}\right)\Delta t_{23},\\ \psi (i) & =R_{c_{1},t_{d}}^{w}{R_{b}^{c}}^{*}\left(\Delta {\bar{p}}_{12}\Delta t_{23}-\Delta {\bar{v}}_{12}\Delta t_{12}\Delta t_{23}\right)\\ \; & \;\;\;-R_{c_{2},t_{d}}^{w}{R_{b}^{c}}^{*}\Delta {\bar{p}}_{23}\Delta t_{12}\\ \; & \;\;\;-\frac{1}{2}R_{e}^{w}\cdot G^{e}\left(\Delta t_{12}\Delta t_{23}^{2}+\Delta t_{12}^{2}\Delta t_{23}\right), \end{array}$$

where ${\left[\cdot \right]}_{\left(:,1:2\right)}$ means the first two columns of the matrix. With *N* keyframes, a linear over-determined system equation $D_{3(N-2)\times 9}\cdot y_{9\times 1}=E_{3(N-2)\times 1}$ with weights for outlier handling can be established to solve $s^{*}$, $\delta \theta_{xy}^{*}$, $\delta b_{a}^{*}$, and ${p_{b}^{c}}^{*}$. Since the accelerometer bias is set to zero when integrating $\Delta {\bar{R}}_{i,i+1}$, $\Delta {\bar{v}}_{i,i+1}$, and $\Delta {\bar{p}}_{i,i+1}$, the final result is $b_{a}^{*}=0_{3\times 1}+\delta b_{a}^{*}=\delta b_{a}^{*}$. The gravity is refined by appending the perturbation, i.e., ${g^{w}}^{*}=R_{e}^{w}\mathrm{Exp}\left(\delta \theta^{*}\right)\cdot G^{e}$. Note that since there are 3(N−2) equations and a 9-dimension unknown vector in the system equation, at least five keyframes are required to solve it.

### 4.3. Updates, Termination, and Velocity Estimation

After each execution of the incremental estimator, the timestamps of subsequent visual measurements are compensated by applying the estimated time offsets, i.e., ${t_{s}^{cam}}^{\prime }=t_{s}^{cam}+t_{d}$. The next estimation will be performed using newly obtained and compensated data.

The incremental estimation will be continuously performed until the estimates get converged. The convergence is judged by using the criteria proposed by [19]. The velocities of all keyframes are estimated uniformly after the parameters are converged. For the old ones, their velocities are computed using the positional relationship of two consecutive keyframes, as shown in (22). For the latest one, since its following keyframes are not determined yet, the velocity propagation equation in (12) is used to calculate its velocity. Once the velocities of all keyframes have been calculated, the scales of camera poses and map points are updated, and the preintegration terms are recomputed to correct the accelerometer bias. The incremental estimation is considered to have been completed after the update and recomputation.

Note that the proposed method does not rely on any prior knowledge about the sensor’s temporal misalignment. If the misalignment is too large, the old uncompensated keyframes will have a bad influence on following executions. We solve this problem by discarding the old keyframes. In particular, if the time offset estimated by (16) is larger than the IMU sampling interval, the time offset will be updated and the system is relaunched. The following approximation and refinement processes will not be performed in this case.

## 5. Visual-Inertial Bundle Adjustment

Note that in the incremental estimation, the gyroscope bias $b_{g}$ and the accelerometer bias $b_{a}$ are assumed to remain constant. This assumption simplifies the process but may slightly reduce the calibration accuracy. To overcome the shortcoming, a visual-inertial bundle adjustment is performed to further correct the spatial-temporal parameters, velocities, and IMU poses and biases. The bundle adjustment can be carried out both globally and locally. When the incremental estimation is completed, global adjustment can be used to refine the accuracy of initial states and extrinsic parameters. The local adjustment is used to combine visual and inertial measurements for visual-inertial odometry.

The bundle adjustment is formulated as a nonlinear optimization problem. We consider the following state vector for an *i*th keyframe as follows:

(25) $$x_{i}=\left[R_{b_{i}}^{w},p_{b_{i}}^{w},v_{b_{i}}^{w},b_{g_{i}},b_{a_{i}},p_{1}^{w},p_{2}^{w},\ldots ,p_{m}^{w}\right],$$

where $p_{k}^{w}\in R^{3}$ is the *k*th map point in the world reference frame observed by the *i*th keyframe. The full state of a nonlinear optimization is defined as follows:

(26) $$X=[x_{l},x_{l+1},\ldots ,x_{n},R_{c}^{b},p_{c}^{b},t_{d}],$$

where *n* and *l* are, respectively, the newest and oldest keyframe indices in an optimized window with size L. The setting of *l* depends on the type of adjustment. For global adjustment, *l* is set to 1. All states starting from the first keyframe are optimized, excluding the position and rotation of the first keyframe as it is regarded as the global reference frame. For local adjustment, *l* is set to n−L+1. The states of the keyframes contained in a local window are accounted.

The nonlinear optimization minimizes both the feature reprojection errors and the IMU preintegration errors, as follows:

(27) $$X^{*}=\underset{X}{\arg\min}\sum_{i=l}^{n}\;\left(\sum_{k}E_{proj}\left(k,i\right)+E_{imu}\left(i-1,i\right)\right),$$

where $E_{proj}\left(k,i\right)$ is the feature reprojection error term for a given matched *k*th map point observed by the *i*th keyframe. $E_{imu}\left(i-1,i\right)$ is the IMU preintegration error term that links keyframe *i* and its previous keyframe i−1.

### 5.1. Feature Reprojection Error

Considering a 3D map point $p_{k}^{w}$ observed by the *i*th keyframe and matched to a 2D image feature, the map point can be transformed into a local camera coordinate frame using Equations (10) and (11) as follows:

(28) $$p_{k}^{c_{i}}=R_{b}^{c}\mathrm{Exp}\left({\tilde{\omega }}_{b_{i}}t_{d}\right){R_{b_{i}}^{w}}^{T}\left(p_{k}^{w}-p_{b_{i}}^{w}+v_{b_{i}}^{w}t_{d}\right)+p_{b}^{c}.$$

Here, we approximate ${\bar{\omega }}_{b_{i}}$ by ignoring the white sensor noise. ${\bar{\omega }}_{b_{i}}$ can thus be written as ${\bar{\omega }}_{b_{i}}\approx {\tilde{\omega }}_{b_{i}}=\omega_{b_{i}}-{\bar{b}}_{g_{i}}-\delta b_{g_{i}}$. Using Equation (28), the feature reprojection error term can be defined as follows:

(29) $$E_{proj}\left(k,i\right)=\rho \left({\left(u_{k}^{i}-\pi \left(p_{k}^{c_{i}}\right)\right)}^{T}\Sigma_{k}\left(u_{k}^{i}-\pi \left(p_{k}^{c_{i}}\right)\right)\right),$$

where $\pi :R^{3}\to \Omega$ is the projection function of pinhole camera model [56]. It transforms a 3D point in the local camera coordinate frame into a 2D point on the image plane. $u_{k}^{i}\in R^{2}$ is the pixel location of the matched feature. $\Sigma_{k}$ is the information matrix associated with the feature detection. ρ is a Huber robust cost function. The states in Equation (29) include the extrinsic spatial and temporal parameters, as well as the IMU pose, IMU velocity, and map points. The Jacobians of reprojection error w.r.t. the states are derived in Appendix A.5.

### 5.2. IMU Preintegration Error

With a slight abuse of notation, we use *i* and *j* to denote two consecutive keyframes for convenience. The IMU preintegration error term $E_{imu}\left(i,j\right)$ is represented as:

(30) $$\begin{array}{cc} E_{imu}\left(i,j\right) & =\rho \left(\left[e_{R}^{T}\;e_{v}^{T}\;e_{p}^{T}\right]\Sigma_{I}{\left[e_{R}^{T}\;e_{v}^{T}\;e_{p}^{T}\right]}^{T}\right)+\rho \left(e_{b}^{T}\Sigma_{b}e_{b}\right),\\ e_{R} & =\mathrm{Log}\left({\left(\Delta {\bar{R}}_{ij}\mathrm{Exp}\left(J_{\Delta {\bar{R}}_{ij}}^{g}\delta b_{g_{i}}\right)\right)}^{T}{R_{b_{i}}^{w}}^{T}R_{b_{j}}^{w}\right),\\ e_{v} & ={R_{b_{i}}^{w}}^{T}\left(v_{b_{j}}^{w}-v_{b_{i}}^{w}-g^{w}\Delta t_{ij}\right)\\ \; & \;\;\;-\left(\Delta {\bar{v}}_{ij}+J_{\Delta {\bar{v}}_{ij}}^{g}\delta b_{g_{i}}+J_{\Delta {\bar{v}}_{ij}}^{a}\delta b_{a_{i}}\right),\\ e_{p} & ={R_{b_{i}}^{w}}^{T}\left(p_{b_{j}}^{w}-p_{b_{i}}^{w}-v_{b_{i}}^{w}\Delta t_{ij}-\frac{1}{2}g^{w}\Delta t_{ij}^{2}\right)\\ \; & \;\;\;-\left(\Delta {\bar{p}}_{ij}+J_{\Delta {\bar{p}}_{ij}}^{g}\delta b_{g_{i}}+J_{\Delta {\bar{p}}_{ij}}^{a}\delta b_{a_{i}}\right),\\ e_{b} & =\delta b_{j}-\delta b_{i}, \end{array}$$

where $\delta b_{j}={\left[{\delta b_{g_{j}}}^{T}\;{\delta b_{a_{j}}}^{T}\right]}^{T}$. $e_{R}$, $e_{v}$, and $e_{p}$ are, respectively, the errors of the preintegrated rotation, velocity, and position. $e_{b}$ is the bias difference between *i* and *j* time instants. $\Sigma_{I}$ and $\Sigma_{b}$ are, respectively, the information matrices of the preintegration and the bias random walk.

## 6. Experiments Furthermore, Discussions

We implement the proposed method based on ORB-SLAM2 [47,48]. The *Tracking* and *Local Mapping* threads of ORB-SLAM2 are used to process camera measurements and output up-to-scale camera poses. The output poses, together with IMU measurements, are sent to our incremental estimator for initialization and calibration. When the incremental estimation is converged, global adjustment is performed to adjust the results and improve accuracy. After that, local adjustment is used to fuse the subsequent camera and IMU measurements to realize the ego-motion estimation and scene reconstruction. Since our focus is on odometry, the *Loop Closure* thread of ORB-SLAM2 is disabled. All the compared methods are also run without loop closure for a fair comparison. The optimized window size L is set to 20. The platform used in the experiments was an Intel CPU i7-4720HQ (8 cores @2.60 GHz) laptop computer with 8GB RAM (Lenovo, Beijing, China).

Performances of the proposed method were evaluated using both synthetic sequences and public real-world datasets. First, we carried out simulation experiments to analyze the robustness performance of the incremental estimator against various IMU sensor noises. The robustness was quantified using errors on the spatial-temporal parameters, gyroscope bias, and accelerometer bias. All of them are defined as scalars for better observing the difference between the estimated result and the ground-truth. The calibrated and the ground-truth rotation matrices are represented in *yaw-pitch-roll* Euler angles. Then, we studied the overall performance of both the incremental estimation and bundle adjustment using real-world experiments. The absolute translational root mean squared error (RMSE) [57] between the estimated trajectory and the ground-truth is used to analyze the overall odometry performance.

### 6.1. Robustness Performance against Various IMU Noises

**Figure 4:**
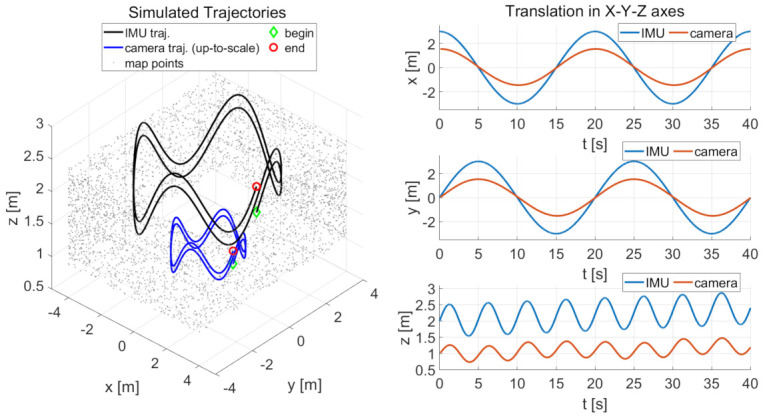
The left subplot shows the parametric IMU trajectory (black curve), up-to-scale camera trajectory (blue curve), and map points (gray dots). The three right subplots are the translation of the camera and IMU poses in x, y, z-axis, with a time offset of 0.1 s. The radius of the circular motion in the x-y plane is 3 m. The amplitude of the vertical sinusoidal motion in the z-axis is (0.5+0.01·t) m. The frequency is 0.2 Hz. The metric-scale of camera poses is customized to 2.0. The green diamond and red circle, respectively, represent the beginning and the end of the trajectory. The simulated sensor moves for 40 s; thus, the total length of the trajectory is 41.59 m.

The robustness performance of the incremental estimator is analyzed using simulation with varying IMU sensor noises and time offsets. We collected data by simulating an IMU that moves along a sinusoidal circle motion. The IMU and camera trajectories are shown in the left subplot of Figure 4. The IMU trajectory is colored in black. The radius of the circular motion in the x-y plane is 3 m, and the amplitude of the vertical sinusoidal motion in the z-axis is (0.5+0.01·t) m with a frequency of 0.2 Hz. The simulated IMU moves last for 40 s; thus the total length of the IMU trajectory is 41.59 m. The up-to-scale camera trajectory is colored in blue. Its poses are generated by transforming the IMU poses using predefined camera-IMU extrinsic spatial parameters (The predefined camera-IMU extrinsic parameters for simulation are set as: $R_{c}^{b}=\left[180.0,0.0,0.0\right]$ deg for rotation and $p_{c}^{b}=\left[0.1,0.04,0.03\right]$ m for translation), while suffering to a predefined metric-scale of 2.0. The translations of the camera and IMU poses are also shown in the right subplots of Figure 4.

**Figure 5:**
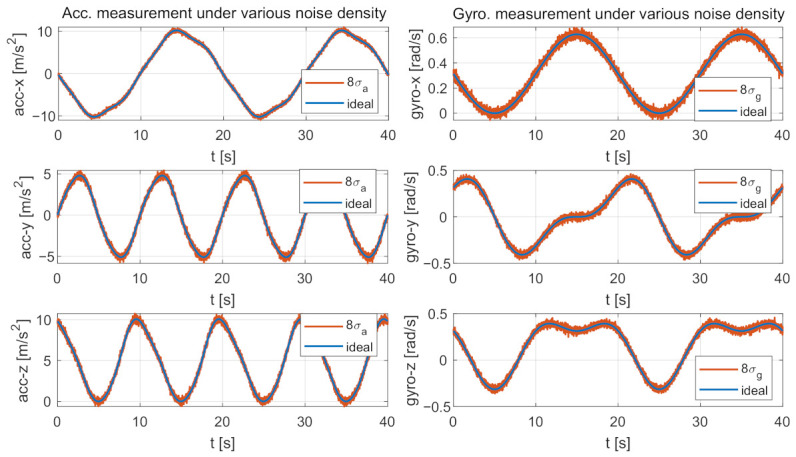
Two examples of the comparison between the noise-free and the noise-affected IMU measurements. The noise-free measurements are generated by computing the analytical derivatives of the parametric trajectory shown in Figure 4. The left subplots compare the noise-free accelerometer measurements with the noise-affected ones. The noise-affected measurements are generated by adding Zero-mean Gaussian noises with densities of $8\sigma_{a}$ to the three axes of the noise-free accelerometer measurements. The right subplots show a similar comparison for gyroscope measurements.

The IMU measurements were generated as follows. First, we prepared noise-free IMU measurements by computing the analytical derivatives of the parametric trajectory. Then, several synthetic noise-affected IMU measurements were generated by adding different intensities of noises to the noise-free measurements. We particularly considered six types of gyroscope-related and accelerometer-related noises, including the measurement noises ($\sigma_{g}$, $\sigma_{a}$), the biases ($b_{g}$, $b_{a}$), and the bias “diffusion” random walk noises ($\sigma_{bg}$, $\sigma_{ba}$). For each type of noise, we designed eight different intensities by starting from a basic zero-mean Gaussian noise model (The basic IMU sensor noises are Gaussian-based noises: gyroscope and accelerometer continuous-time noise densities: $\sigma_{g}=0.00017\;\mathrm{rad}/\left(s\sqrt{\mathrm{Hz}}\right)$, $\sigma_{a}=0.002\;m/\left(s^{2}\sqrt{\mathrm{Hz}}\right)$. Constant biases: $b_{g}=\left[-0.0023,0.0249,0.0817\right]\;\mathrm{rad}/s$, $b_{a}=\left[-0.0236,0.1210,0.0748\right]\;m/s^{2}$. Bias “diffusion” random walk noise densities: $\sigma_{bg}=0.00002\;\mathrm{rad}/\left(s^{2}\sqrt{\mathrm{Hz}}\right)$, $\sigma_{ba}=0.003\;m/\left(s^{3}\sqrt{\mathrm{Hz}}\right)$. We highlight that these basic noises are similar to the ground-truth values provided by EuRoC dataset [37], therefore they are meaningful in practical application.) As a result, we get 48 (6 × 8) noise-affected IMU data sequences. Each sequence is affected by one type of noise with particular intensity. Figure 5 exemplifies two synthetic noisy IMU measurements compared with their corresponding noise-free values. The simulated sampling rates of the camera and IMU are 20 Hz and 200 Hz, respectively.

**Figure 6:**
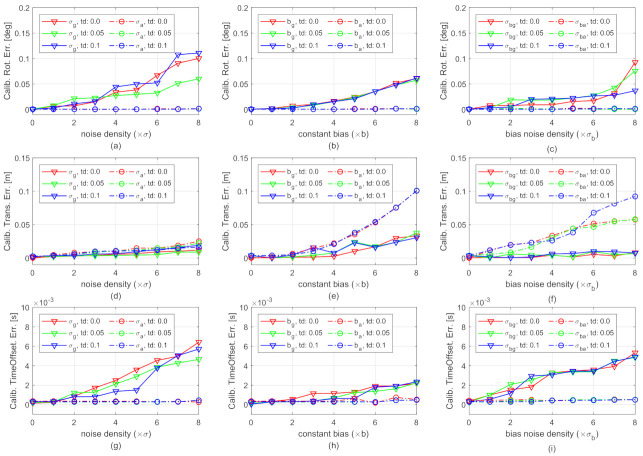
Spatial-temporal calibration errors in the presence of various gyroscope and accelerometer noises. The vertical axes of (**a**–**c**), (**d**–**f**), and (**g**–**i**) subplots are, respectively, the calibration error of extrinsic camera-IMU rotation, translation, and time offset. The horizontal axes of the subplots in the first column, the second column, and the third column are, respectively, the intensities of measurement noise density, constant bias, and bias “diffusion” random walk noise density. For instance, the labels “0” in all the horizontal axes represent the ideal noise-free measurements. The label “4” in the horizontal axis of (**a**) means the measurement noise density is customized to $4\sigma_{g}$ (for evaluating the robustness on gyroscope measurement noise, of which results are shown in solid curves) or $4\sigma_{a}$ (for evaluating the robustness on accelerometer measurement noise, of which results are shown in dash-dot curves), while the other noises are customized to zeros. **Best viewed in color**.

Figure 6 shows the camera-IMU spatial-temporal calibration errors in the presence of various gyroscope and accelerometer noises. The horizontal axes of the subplots are the intensities of sensor noises. The vertical axes are calibration errors. The solid curves and dash-dot curves are, respectively, the results of the gyroscope-related and accelerometer-related noises. The different colors show the results of different time offsets: red-0 ms, green-50 ms, blue-100 ms. The results in Figure 6 are the median over 25 tests. Since the proposed method does not rely on any prior knowledge, the spatial-temporal parameters are initially set to 0 in the tests.

The results in Figure 6a–c,g–i show that both the calibration results of rotation and time offset are satisfying. For example, all the rotation errors are smaller than $0.15^{\circ }$. When the gyroscope noise density and bias noise density are smaller than $7\sigma_{g}$ and $7\sigma_{bg}$, the calibrated time offsets are smaller than the simulated IMU sampling period (5 ms). The results also show that the influence of changing accelerometer noises on the rotation and time offset calibration is negligible. This is reasonable since only the gyroscope-related parameters are involved in the first process of the incremental estimation (see Equation (15)).

Figure 6d–f compare the translation errors of calibration under different noises. As shown in Figure 6d, the growth of the translation error is moderate with the increase of noise densities of both gyroscope and accelerometer measurement. The error remains less than 0.025 m even when the measurement noise density increases to $8\sigma_{a}$ and $8\sigma_{g}$. The moderate growth implies that the translational calibration is robust to IMU measurement noise. The solid curves in Figure 6f show that the translational calibration is robust to gyroscope bias noise. The curves in Figure 6e and the dash-dot curves in Figure 6f show that the translational calibration may get worse when the constant biases or the accelerometer bias noise density are large. However, the results are still satisfying, i.e., 0.025 m errors, when the constant biases are smaller than $6b_{g}$ and $4b_{a}$, and the accelerometer bias noise is smaller than $3\sigma_{ba}$.

Figure 6 also shows that the calibration errors are not significantly influenced by changing time offsets, as the curves with the same type but different colors in each subplot do not have significant differences. Detailed calibration errors of spatial-temporal parameters and IMU biases in the presence of all different types of IMU noises with their first intensities are provided in Table 1. The results also show that the errors are not much different w.r.t. varying time offsets. The observation is further analyzed using real-world datasets.

### 6.2. Overall Performance of Both the Incremental Estimation and the Bundle Adjustment on Public Dataset

#### 6.2.1. Dataset

The overall performance of both the incremental estimation and the bundle adjustment is evaluated using the real-world EuRoC dataset [37]. The dataset contains eleven sequences. Five of them were recorded in a large machine hall (denoted as MH_01 to MH_05), and the other six were recorded in a room using the Vicon motion capture system (denoted as V1_01 to V1_03 and V2_01 to V2_03). By considering the illumination, texture, motion velocity, and motion blur, the dataset is classified into *easy*, *medium*, and *difficult* grades. The ground-truth gyroscope bias, accelerometer bias, spatial parameters (The ground-truth extrinsic spatial parameters provided by the dataset were calibrated by the *Kalibr* [9,10,11,12] toolbox, with [89.147953, 1.476930, 0.215286] deg in yaw, pitch, roll directions for $R_{c}^{b}$, and [−0.021640, −0.064677, 0.009811] m in x, y, z directions for $p_{c}^{b}$), IMU body velocities, and flying trajectories are given in the dataset. The original dataset does not have time delays. We include them by further adding millisecond values to the image timestamps. There is a fixed time offset between the IMU and camera measurements after the addition. To verify the capability of on-the-fly initialization, the start time of the time-shifted sequence is randomly selected in each test, so that the proposed and compared methods can start without any prior information about when the platform begins to fly.

#### 6.2.2. Robustness Performance Concerning Various Time Offsets

In this experiment, we compared the robustness performance of different methods when concerning various time offsets. In particular, the predefined time offset ranges from −100 to 100 ms with a step of 5 ms. The accuracy of calibrated extrinsic parameters and the sensor ego-motion estimation of visual-inertial odometry were both evaluated using the MH_03 sequence, which contains fast motion and significant illumination change.

**Figure 7:**
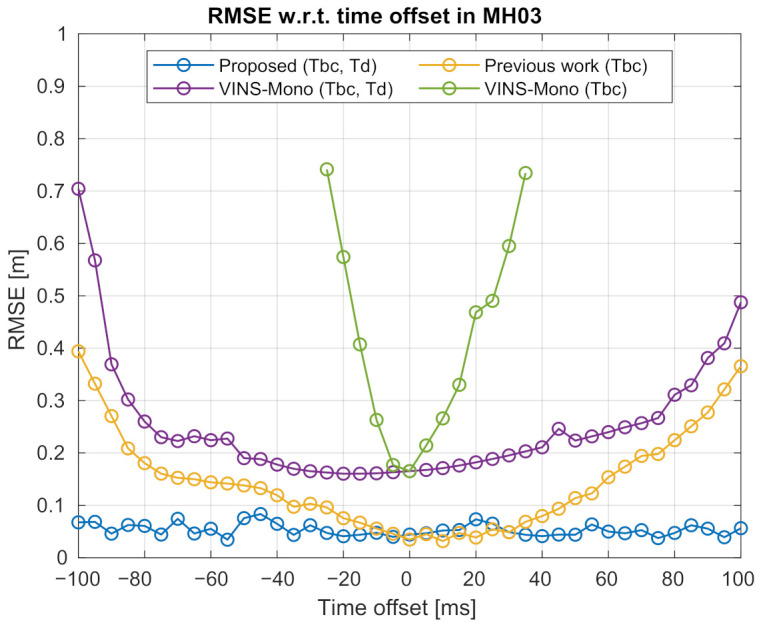
Comparison of the trajectory accuracy under various time offsets using the MH_03 sequence. The x-axis denotes the various time offsets. The y-axis shows the absolute translational root mean squared error (RMSE) of the estimated trajectory. The curves shown in the figure are, respectively, “Proposed (Tbc, Td)”—Results of the proposed method, which can perform simultaneous spatial-temporal calibration; “Previous work (Tbc)”—Results of our previous work [36], which only performs spatial calibration; “VINS-Mono (Tbc, Td)”—Results of VINS-Mono [40], which can perform simultaneous spatial-temporal calibration; “VINS-Mono (Tbc)”—Results of VINS-Mono, which only performs spatial calibration. **Best viewed in color**.

As shown in Figure 7 where the median RMSEs of the estimated trajectories over 25 tests are presented, the time-shifted sequences are tested with VINS-Mono [40], our previous method [36], and the proposed method.

VINS-Mono (https://github.com/HKUST-Aerial-Robotics/VINS-Mono, accessed on 14 February 2021) is a popular open-source VIO system that can perform online spatial-temporal calibration. It provides three configurations for spatial calibration, i.e., “*with extrinsic parameters*”, “*have initial extrinsic guess*”, and “*no extrinsic parameters*”, as well as one configuration for temporal calibration, i.e., “*estimate time offset*”. For the “*no extrinsic parameters*” configuration, the public source code integrates a method called automatic estimator initialization and online extrinsic spatial calibration [18]. For the “*estimate time offset*” configuration, the code integrates a method developed in [22]. In our experiments, VINS-Mono is launched in all these configurations for inspection. The purple curve in Figure 7 is the results when VINS-Mono is launched under “*no extrinsic parameters*” and “*estimate time offset*” configurations for fairly comparing with the proposed method. In this case, the extrinsic spatial and temporal parameters can be continuously optimized by the bundle adjustment as new measurements arrive. The green curve in the same figure shows the result when VINS-Mono is launched under the single “*no extrinsic parameters*” configuration while the temporal calibration ability is disabled. Note that all these methods are launched without given any initial guess about extrinsic spatial or temporal parameters. In the experiments, we find out that the results of “VINS-Mono (Tbc)” have significantly large errors when the time offset surpasses 35 or −25 ms. These results are not plotted for limiting the range of the y-axis. The performance of “VINS-Mono (Tbc, Td)” is much better than “VINS-Mono (Tbc)” as the latter one does not calibrate the temporal parameters.

Our previous method can calibrate extrinsic spatial parameters online without considering the temporal parameters. The results of using our previous method are shown in the yellow curve with a legend of “Previous work (Tbc)”. This shows that the previous work can accurately estimate the sensor trajectory only when the time offset is within −10 to 35 ms. The trajectory accuracy of the previous work deteriorates remarkably when the time offset increases. The average RMSE of the previous work is 0.146 m. The blue curve is the result of the method proposed in this work. The trajectory accuracy of the proposed method is better than our previous work, especially when the time offset surpasses 35 or −10 ms. The proposed method also achieves much lower RMSE in all the time offsets compared to the “VINS-Mono (Tbc, Td)” method. The trajectory estimated by the proposed method exhibits consistent accuracy under different time offsets, with an average error of 0.035 m. On the contrary, although “VINS-Mono (Tbc, Td)” has consistent accuracy when the time offset is between −75 and 75 ms, its performance deteriorates remarkably when the time offset increases. The reason is that the authors used image feature velocity for modeling and compensating the temporal misalignment. The usage is under the assumption that in a short period, an image feature moves at a constant velocity on the image plane. However, when the time offset is large, this assumption could be violated. The feature velocity cannot compensate well for large temporal misalignment.

**Figure 8:**
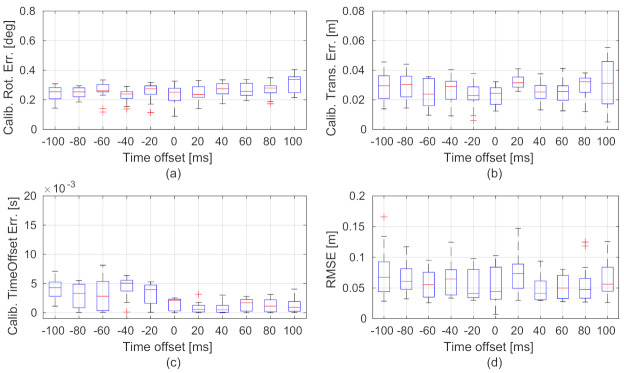
Statistical visualizations of the spatial-temporal calibration errors (**a**–**c**) and trajectory accuracy (**d**) under various time offsets using the MH_03 sequence. The x-axes of the subplots show the various time offsets. The box plots are the results of 25 tests using randomly selected start times.

The statistical visualizations of the spatial-temporal calibration errors and trajectory accuracy of our method under various time offsets are, respectively, shown in Figure 8a–d. The results show that the calibration errors and trajectory accuracy do not have much difference under various time offsets. The performance of extrinsic calibration and ego-motion estimation of the proposed method is robust to asynchronous visual and inertial measurements.

#### 6.2.3. Convergence Performance

**Figure 9:**
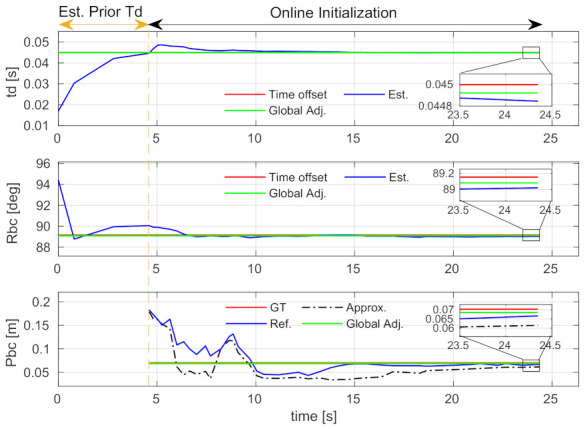
Comparison of the calibrated spatial and temporal parameters using the V2_01 sequence. The section labeled by “Est. Prior Td” denotes a stage where we update the time offset and relaunch the system. Abbreviations: Est.—Estimated; Global Adj.—Global adjustment; GT—Ground-truth; Approx.—Approximation; Ref.—Refinement. **Best viewed in color**.

**Figure 10:**
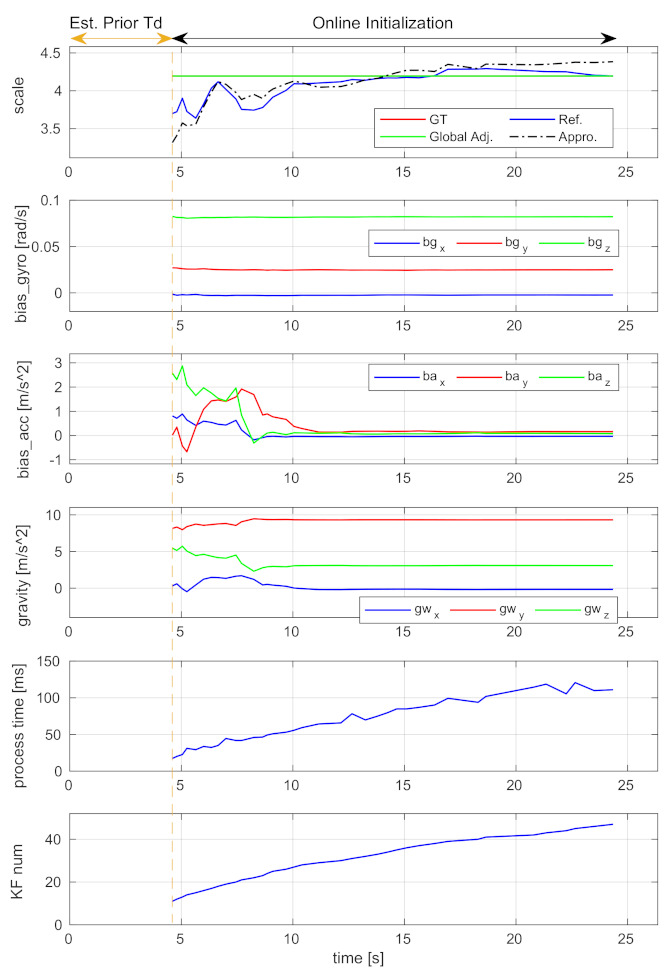
The scale, gyroscope bias (bias_gyro), accelerometer bias (bias_acc), gravity vector, processing time expended by each execution, and keyframe number (KF num) using the V2_01 sequence.

The convergence performance of spatial-temporal calibration and initial value estimation is studied using the V2_01 sequence with a 45 ms time offset. The time-varied estimation results of the spatial-temporal parameters and the initial states are, respectively, shown in Figure 9 and Figure 10. Note that, as described in Section 4.3, if a new time offset estimated by Equation (16) is larger than the IMU sampling interval, our system will be relaunched. The sections before the vertical dash lines in the figures show a relaunching action. These sections are denoted as “Est. Prior Td”. Since the approximation and refinement processes are skipped in these sections, the result of translation and all results of the initial states are not available.

The “Online Initialization” sections after the vertical dash lines are the relaunched results. The diagrams of the estimated time offset, extrinsic rotation, and gyroscope bias show that these parameters can converge to stable values within a few seconds, and the stable values are very close to the ground-truth. The proposed incremental estimator can effectively estimate them. The diagrams of the translation and scale show that the refined results (i.e., blue curves) are better than the approximated ones (i.e., black dash-dot curves). The results are even better if the global adjustment is performed (i.e., green curves). These indicate that the proposed incremental estimator can estimate the spatial-temporal parameters from coarse-to-fine, and the bundle adjustment can effectively improve the estimation.

One thing to note is that the curves of accelerometer bias and gravity exhibit severe oscillation in the first few seconds. This is because the platform did not have enough excitation in the beginning. The values become estimable with the arrival of more keyframes.

#### 6.2.4. Velocity Estimation

**Figure 11:**
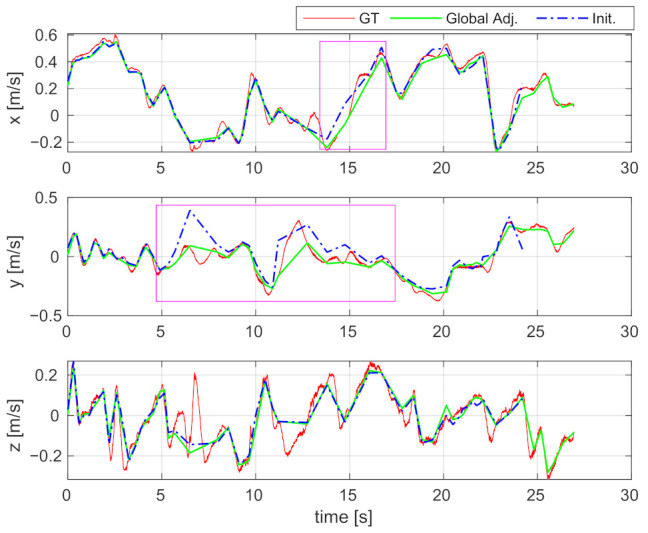
Comparison of the estimated IMU body velocities using the V2_01 sequence of the EuRoC dataset. Red curve: Ground-truth velocity (GT); Blue dash-dot curve: The velocity estimated by online initialization (Init.); Green curve: The velocity corrected by global adjustment (Global Adj.). **Best viewed in color**.

The estimated IMU body velocities are shown in Figure 11. Since the estimated results and ground-truth are expressed in different coordinate systems, we transform the estimated results to help readers better compare with the ground-truth. The estimated velocities in the diagrams (i.e., blue dash-dot curves) are almost consistent with the ground-truth (i.e., red curves), indicating that the velocities are well estimated by the proposed incremental estimator. In the sections marked by pink boxes, the results of global adjustment are even closer to the ground-truth, which indicates that the accuracy of velocity estimation can be further improved by performing a global adjustment. The RMSE of the estimated velocities decreased from 0.093 m/s to 0.046 m/s by applying global adjustment. The consistency between the estimated results and the ground-truth also shows that the metric-scale can be correctly estimated, since otherwise, the magnitude of estimated velocity would differ from the ground-truth.

#### 6.2.5. Computational Complexity Analysis

In Figure 10, the keyframe number and the processing time expended by each execution of the incremental estimator are plotted. It shows that the processing time is approximately linear to the number of keyframes, indicating that the proposed incremental estimator has linear time complexity. To analyze the increased computation load on bundle adjustment due to the calculation of spatial-temporal parameters, we perform the global and local bundle adjustments with or without optimizing these parameters. The execution times are shown in Table 2. The global adjustment takes about 10 s and the local adjustment takes about 1.5 s for optimization. In detail, by further calculating the extrinsic spatial-temporal parameters, the global adjustment takes an extra 2.709 s and the local adjustment takes an extra 0.087 s. Note that since the two adjustments are middle-scale or large-scale optimization problems as they optimize thousands of variables, they are usually executed in separate threads in practice. In addition, the global adjustment is only performed one time when the incremental estimation is completed, and the local adjustment is performed only when a new keyframe is detected. Therefore, the extra time cost will not affect the real-time pose tracking of visual-inertial odometry.

#### 6.2.6. Accuracy on the Whole Dataset

We also studied the overall performance using the whole dataset. The various time offsets are preset to 0 ms, 50 ms, and 100 ms for comparison. Each sequence is tested 25 times under each time offset. VINS-Mono is launched under “*no extrinsic parameters*” and “*estimate time offset*” configurations. Feng’s method [44] is also compared. Table 3 shows the estimation errors of the spatial-temporal parameters and the absolute translational RMSE of the estimated trajectories. The average rotational and translational errors using the parameters calibrated by our method are, respectively, $0.155^{\circ }$ and 0.016 m, which are better than VINS-Mono ($0.276^{\circ }$ and 0.022 m) and Feng’s method ($0.559^{\circ }$ and 0.018 m). The average temporal error using the parameter calibrated by our method is 0.773 ms. The value seems less accurate than VINS-Mono (0.133 ms). The reason is VINS-Mono treats the time offset as a vision factor by interpolating the location of features on the image plane, which exhibits better accuracy on temporal calibration than ours. Despite the low accuracy in temporal calibration, the trajectory RMSEs of our method are smaller than VINS-Mono and Feng’s method on most sequences, implying that it has a better performance on sensor pose estimation. The dominance of our method owes to its better accuracy in spatial calibration.

Table 4 shows the detailed errors of the estimated gyroscope biases, accelerometer biases, and velocities. The mean, standard deviation, and the maximum values are the statistical results over 75 tests (There are three types of predefined time offsets, and each sequence is tested 25 times at each time offset. Thus, a sequence has tested a total of 75 times.). The results indicate that the IMU biases can be accurately estimated. The maximum errors for gyroscope bias and accelerometer bias are, respectively, 0.00158 rad/s and 0.1219 m/$s^{2}$. The maximum error for velocity is 0.0861 m/s, which is less than a few centimeters per second even for the difficult sequences, e.g., V1_03, V2_03, MH_04, and MH_05 sequence.

### 6.3. Real Sensor Experiments

The Intel RealSense Depth Camera D435i (https://www.intelrealsense.com/depth-camera-d435i/, accessed on 14 February 2021) was used in the real sensor experiment. This sensor contains a three-axis accelerometer with a sample rate of 250 Hz, a three-axis gyroscope with the sample rates of 200 or 400 Hz, two global infrared cameras, and one rolling shutter RGB camera. In this experiment, we used the accelerometer, the gyroscope, and the left infrared camera. The infrared camera was launched at 15 Hz with a resolution of 640 × 480. Since the accelerometer and the gyroscope run at different rates, the gyroscope was launched at 400 Hz and its measurements were down-sampled and linearly interpolated to synchronize with the accelerometer. The synchronized accelerometer and gyroscope data constitute the six-axis IMU measurements, with a rate of 250 Hz. According to official documents, the inertial sensors and cameras can be triggered to synchronize. However, we find that actually there is a noticeable time offset between IMU and camera measurements, and its value is related to the exposure time of the camera.

**Figure 12:**
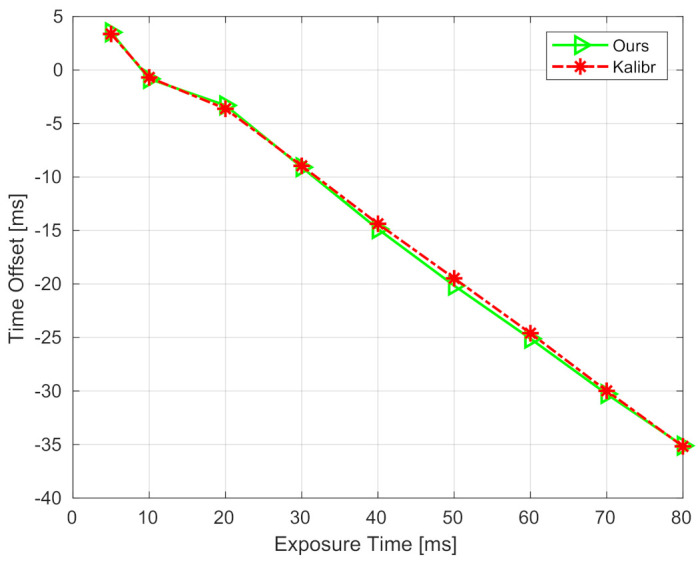
Calibrated time offsets under different exposure times of RealSense-D435i.

To evaluate the robustness performance of the proposed method on real sensors, we set nine different camera exposure times, from 5 to 80 ms. For each exposure time, five sequences facing toward a checkerboard were collected for offline calibration, and ten sequences in a living room were collected for testing the proposed method. In this experiment, the state-of-art toolbox Kalibr [9,10] was adopted to offline-calibrate the extrinsic spatial-temporal parameters. The offline-calibrated results are regarded as ground-truth values and used to compare with the proposed method. The comparison of calibrated time offsets under different exposure times is shown in Figure 12. It can be seen that, first, the time offset is approximately linear to the exposure time, with a slope of about −0.5. The time offset is about zero when the exposure time is set to 10 ms. This means that one can obtain well-aligned IMU and camera measurements when setting the exposure time to 10 ms. However, as the exposure time increases, the estimated time offset becomes negative, and its absolute value increases. The reason is that the middle of exposure time is recorded as the timestamp of an image. Second, the time offsets calibrated by our method (i.e., green curve) are close to those calibrated by Kalibr (i.e., red curve). Note that Kalibr is an offline method, which consumes more time and computation resources than ours. The comparison indicates that the proposed method is comparable to offline methods. The calibration errors of extrinsic rotation and translation are also shown in Table 5. It can be noticed that all the errors of rotation and translation are, respectively, smaller than 0.45 degrees and 0.017 m, even though the exposure time increases. These results illustrate that the proposed method can accurately calibrate the extrinsic spatial and temporal parameters, and it is robust to various exposure times when applying to a real visual-inertial sensor.

## 7. Conclusions and Future Work

This paper presented an online initialization and spatial-temporal calibration method for monocular visual-inertial odometry. The method used two short-term motion interpolation and an incremental estimator for online initialization and spatial-temporal calibration, and used bundle adjustment to fuse the camera and IMU measurements and adjust the estimated system states. The method does not rely on any prior knowledge about the spatial or temporal configuration. Thus, it is suitable for the specification-free and asynchronous VIO sensors. The performance of the proposed method was evaluated using both synthetic sequences and public datasets. The results showed that the initial states and spatial-temporal parameters could be accurately estimated and converge in a short time, and they could be well corrected by the introduced bundle adjustment. The proposed method is concluded to have a competitive accuracy compared with the existing methods.

One shortcoming of this work is that the proposed method is particularly designed for monocular VIOs. In practical applications, there are various types of odometers, like stereo/duo visual-inertial odometry, Lidar-visual-inertial odometry, Encoder-visual-inertial odometry, GPS-visual-inertial odometry, etc. In the future, we will extend the proposed method by applying it to other types of odometers.

## Figures and Tables

**Table 1:** Errors of extrinsic spatial-temporal parameters and IMU biases in the presence of all different types of IMU noises.

Time Offset	$e_{\mathrm{rot}}$	$e_{\mathrm{trans}}$	$e_{\mathrm{td}}$	$e_{\mathrm{bg}}$	$e_{\mathrm{ba}}$
(ms)	(deg)	(m)	(ms)	(rad/s)	(m/s${}^{2}$)
0	0.010	0.014	1.170	0.836 × 10${}^{-4}$	0.853 × 10${}^{-2}$
50	0.015	0.011	1.303	1.026 × 10${}^{-4}$	0.941 × 10${}^{-2}$
100	0.021	0.012	1.503	1.024 × 10${}^{-4}$	1.012 × 10${}^{-2}$

**Table 2:** Execution Times of Global and Local Adjustments With or Without Optimizing the Spatial-Temporal Parameters ${}^{1}$.

Global Adj. with Ext. Opt. (s)	Global Adj. without Ext. Opt. (s)	Local Adj. with Ext. Opt. (s)	Local Adj. without Ext. Opt. (s)
11.834	9.125	1.514	1.427

^1^ All the results are the median over 25 tests on the V2_01 sequence. Abbreviations: Global Adj.—Global adjustment; Local Adj.—Local adjustment; Ext. Opt. — Extrinsic Optimization; with Ext. Opt.—Adjustment includes optimizing the extrinsic spatial-temporal parameters; without Ext. Opt.—Adjustment without optimizing the extrinsic spatial-temporal parameters.

**Table 3:** Spatial-Temporal Calibration Errors and Keyframe Trajectory Accuracy on the EuRoC Dataset ${}^{1}$.

		VINS-Mono [40]				Feng et al.				Ours			
	time offset	$e_{\mathrm{rot}}$	$e_{\mathrm{trans}}$	$e_{\mathrm{td}}$	RMSE	$e_{\mathrm{rot}}$	$e_{\mathrm{trans}}$	$e_{\mathrm{td}}$	RMSE	$e_{\mathrm{rot}}$	$e_{\mathrm{trans}}$	$e_{\mathrm{td}}$	RMSE
	(ms)	(deg)	(m)	(ms)	(m)	(deg)	(m)	(ms)	(m)	(deg)	(m)	(ms)	(m)
*V1_01	0	0.285	0.018	0.291	0.071	0.583	0.022	0.150	0.073	0.099	0.008	0.001	**0.047**
	50	0.286	0.019	0.284	0.075	0.588	0.023	0.210	0.073	0.136	0.008	0.836	**0.043**
	100	0.235	0.019	0.220	0.103	0.577	0.022	0.150	0.077	0.247	0.033	0.244	**0.064**
*V1_02	0	0.327	0.018	0.059	0.095	0.563	0.019	0.090	0.118	0.128	0.011	0.026	**0.025**
	50	0.312	0.013	0.034	0.092	0.559	0.019	0.100	0.116	0.086	0.003	0.082	**0.022**
	100	0.363	0.011	0.053	0.192	0.569	0.021	0.100	0.143	0.154	0.015	2.372	**0.047**
*V1_03	0	0.220	0.012	0.095	0.141	0.507	0.013	0.330	0.118	0.165	0.012	0.340	**0.012**
	50	0.180	0.014	0.005	0.153	0.508	0.016	0.330	0.121	0.276	0.018	3.194	**0.046**
	100	0.229	0.016	0.008	0.192	0.513	0.014	0.390	0.093	0.194	0.023	2.695	**0.053**
*V2_01	0	0.295	0.020	0.152	0.065	0.491	0.023	0.330	0.099	0.183	0.017	1.009	**0.019**
	50	0.259	0.020	0.174	0.068	0.457	0.025	0.290	0.088	0.145	0.002	0.297	**0.014**
	100	0.291	0.020	0.029	0.070	0.513	0.022	0.360	0.082	0.132	0.012	0.279	**0.034**
*V2_02	0	0.353	0.012	0.123	0.134	0.553	0.020	0.090	0.099	0.065	0.010	0.016	**0.028**
	50	0.344	0.012	0.135	0.120	0.558	0.020	0.090	0.089	0.116	0.010	0.174	**0.041**
	100	0.329	0.012	0.031	0.140	0.558	0.020	0.090	0.100	0.220	0.014	1.751	**0.059**
*V2_03	0	0.429	0.011	0.271	0.150	0.633	0.015	0.090	0.135	0.177	0.027	0.403	**0.091**
	50	0.455	0.011	0.262	0.337	0.626	0.015	0.040	0.234	0.203	0.012	0.459	**0.079**
	100	0.409	0.041	0.010	0.252	0.633	0.014	0.040	0.233	0.206	0.011	1.368	**0.096**
*MH_01	0	0.168	0.021	0.068	0.134	0.501	0.018	0.160	0.080	0.203	0.009	1.842	**0.022**
	50	0.184	0.021	0.014	0.152	0.505	0.015	0.120	0.119	0.143	0.011	0.125	**0.054**
	100	0.168	0.023	0.155	0.158	0.481	0.014	0.120	0.111	0.082	0.019	0.107	**0.027**
*MH_02	0	0.193	0.016	0.042	0.297	0.621	0.014	0.290	0.082	0.125	0.028	0.596	**0.021**
	50	0.256	0.018	0.020	0.253	0.624	0.014	0.340	0.086	0.280	0.016	0.225	**0.029**
	100	0.306	0.023	0.054	0.220	0.634	0.015	0.210	0.074	0.316	0.013	0.010	**0.033**
*MH_03	0	0.323	0.024	0.184	0.165	0.619	0.022	0.010	0.161	0.251	0.024	2.158	**0.030**
	50	0.325	0.025	0.168	0.224	0.627	0.024	0.050	0.133	0.034	0.011	2.357	**0.038**
	100	0.340	0.022	0.054	0.488	0.607	0.020	0.090	0.173	0.338	0.031	0.948	**0.046**
*MH_04	0	0.211	0.076	0.113	0.239	0.554	0.019	0.110	0.197	0.044	0.019	0.002	**0.153**
	50	0.198	0.033	0.052	0.285	0.521	0.013	0.170	**0.178**	0.109	0.029	0.313	0.240
	100	0.218	0.025	0.347	0.316	0.512	0.018	0.030	**0.143**	0.090	0.023	1.279	0.215
*MH_05	0	0.187	0.029	0.237	0.184	0.605	0.013	0.090	**0.162**	0.107	0.016	0.472	0.204
	50	0.201	0.029	0.211	0.248	0.509	0.010	0.200	**0.207**	0.118	0.012	0.437	0.254
	100	0.236	0.028	0.101	0.296	0.552	0.017	0.170	**0.205**	0.104	0.026	0.616	0.214

${}^{1}$ All the results of our method and VINS-Mono are the median of 25 tests on each sequence of the EuRoC dataset using randomly selected start times. Abbreviations: $e_{rot}$—error of the extrinsic rotation, $e_{trans}$—error of the extrinsic translation, $e_{td}$—error of the estimated time offset. The values colored in red, green, blue, and bold black are, respectively, the best result of the extrinsic rotation, extrinsic translation, time offset, and trajectory accuracy.

**Table 4:** Errors of the Estimated Initial Values on the EuRoC Dataset ${}^{1}$.

	Bias_Gyro (Rad/s)$\cdot 10^{-3}$			Bias_acc (m/$s^{2}$)$\cdot 10^{-2}$			Velocity (m/s)$\cdot 10^{-2}$		
	Mean	StaDev	Max	Mean	StaDev	Max	Mean	StaDev	Max
V1_01	0.56	0.09	0.66	4.19	0.77	4.30	3.33	1.26	5.97
V1_02	0.52	0.21	1.09	3.48	0.96	5.16	3.35	1.74	6.29
V1_03	1.26	0.10	1.44	6.90	1.18	10.33	5.76	1.08	7.42
V2_01	0.50	0.16	0.84	3.95	1.43	5.85	1.34	0.69	3.46
V2_02	1.16	0.25	1.58	7.17	2.32	12.19	2.01	1.07	8.04
V2_03	0.78	0.12	1.02	6.35	1.20	10.47	2.54	1.32	7.64
MH_01	0.70	0.26	1.30	4.34	1.46	6.19	3.05	1.47	6.72
MH_02	0.32	0.15	0.51	3.81	1.46	5.98	2.17	1.07	4.42
MH_03	0.60	0.18	0.83	3.04	0.85	4.99	4.72	2.23	6.47
MH_04	0.44	0.05	0.50	4.08	0.75	6.04	5.20	1.39	8.61
MH_05	0.33	0.07	0.44	4.57	1.27	7.44	4.27	2.04	8.03

^1^ Note that the time offset was set to 0 ms, 50 ms, and 100 ms. Each sequence was performed with a total of 75 tests (25 tests × 3 types of time offset). Abbreviations: bias_gyro—gyroscope bias, bias_acc—accelerometer bias, staDev—standard deviation.

**Table 5:** Calibration Errors of Extrinsic Spatial Parameters under Different Exposure Times ${}^{1}$.

Exp. Time	5 ms	10 ms	20 ms	30 ms	40 ms	50 ms	60 ms	70 ms	80 ms
$e_{rot}$ /deg	0.355	0.429	0.418	0.402	0.383	0.378	0.242	0.388	0.308
$e_{trans}$ /m	0.012	0.006	0.003	0.013	0.012	0.016	0.015	0.008	0.017

^1^ All the results are the median of ten sequences captured by RealSense-D435i. Abbreviations: *e_rot_*—error of the extrinsic rotation, *e_trans_*—error of the extrinsic translation.

## Appendix A

### Appendix A.1. Preliminaries

The background geometric concepts used in Section 4.2 and the remaining part of this Appendix are as follows.

#### Appendix A.1.1. First-Order Approximation

The exponential map of a rotational vector ϕ∈so(3) is equivalent to a standard matrix exponential (i.e., Rodrigues’ rotation formula):

(A1) $$\exp\left(\phi^{\wedge }\right)=I+\frac{\sin(∥\phi ∥)}{∥\phi ∥}\phi^{\wedge }+\frac{1-\cos(∥\phi ∥)}{{∥\phi ∥}^{2}}{\left(\phi^{\wedge }\right)}^{2}.$$

A first-order approximation of the exponential map is:

(A2) $$\exp\left(\phi^{\wedge }\right)\approx I+\phi^{\wedge }.$$

#### Appendix A.1.2. Adjoint Property

Given a Lie element $\tilde{\phi }=\mathrm{Log}\left(\tilde{R}\right)$ and a rotation R, the adjoint property is:

(A3) $$R\tilde{R}R^{T}=\exp\left(R{\tilde{\phi }}^{\wedge }R^{T}\right)=\exp\left({\left(R\tilde{\phi }\right)}^{\wedge }\right)=\mathrm{Exp}\left(R\tilde{\phi }\right).$$

#### Appendix A.1.3. BCH Linear Approximation

The BCH (Baker–Campbell–Hausdorff [58]) linear approximation for $\phi_{1}$ and $\phi_{2}$ in the Lie algebra of a Lie group is:

(A4) $$\mathrm{Log}\left(\mathrm{Exp}\left(\phi_{1}\right)\mathrm{Exp}\left(\phi_{2}\right)\right)\approx \left\{\;\;\begin{array}{c} J_{l}^{-1}\left(\phi_{2}\right)\phi_{1}+\phi_{2},\;\;\mathrm{if}\;\;\phi_{1}\;\;\mathrm{is}\;\;\mathrm{small},\\ J_{r}^{-1}\left(\phi_{1}\right)\phi_{2}+\phi_{1},\;\;\mathrm{if}\;\;\phi_{2}\;\;\mathrm{is}\;\;\mathrm{small}, \end{array}\right.$$

where $J_{l}^{-1}\left(\cdot \right)$ and $J_{r}^{-1}\left(\cdot \right)$ are, respectively, the inverses of left-Jacobian matrix $J_{l}\left(\cdot \right)$ and right-Jacobian matrix $J_{r}\left(\cdot \right)$. The additive operation of a small perturbation δϕ on Lie algebra can therefore be approximated as follows:

(A5) $$\begin{array}{cc} \mathrm{Exp}(\phi +\delta \phi ) & \approx \mathrm{Exp}(J_{l}\left(\phi \right)\delta \phi )\mathrm{Exp}\left(\phi \right)\\ \; & \approx \mathrm{Exp}\left(\phi \right)\mathrm{Exp}(J_{r}\left(\phi \right)\delta \phi ) \end{array}$$

### Appendix A.2. Gauss–Newton Algorithm

The proposed bundle adjustment considering the time offset in (27) is essentially a least-squares non-convex function, which can be rewritten as a differentiable object function J(x). Our goal is to determine the optimum design parameter $\hat{x}$, that minimizes the objective function:

(A6) $$\hat{x}=\underset{x\in M}{\arg\min}J\left(x\right),$$

where the variables belong to some manifold M. A typical technique to minimize this expression is Gauss–Newton method [55], which iteratively solves the Gauss–Newton update equation on an operating point $x_{op}$ to find the value of δx, i.e., a “small" change to $x_{op}$, and then uses the δx to update $x_{op}$. The method iterates until the change becomes sufficiently small.

The Gauss–Newton update equation is defined as:

(A7) $$\left(H^{T}W^{-1}H\right)\delta x^{*}=H^{T}W^{-1}e\left(x_{op}\right)$$

with

(A8) $$H=-\frac{\partial e(x)}{\partial x}{|}_{x_{op}},$$

where $W^{-1}$ is the block-tridiagonal information matrix. The jacobian of errors w.r.t variables highly depends on the updating manner of the variables. In the following sections, we first define the updating rules of variables, and then derive the Jacobians of different errors.

### Appendix A.3. State Update

We define the small change δx as an element of the tangent space at $x_{op}$. The update equations of the system states are as follows:

(A9) $$\begin{array}{cc} R_{b_{i}}^{w}\leftarrow R_{b_{i}}^{w}\mathrm{Exp}\left(\delta \phi_{b_{i}}\right), & R_{c}^{b}\leftarrow R_{c}^{b}\mathrm{Exp}\left(\delta \phi_{c}^{b}\right),\\ p_{b_{i}}^{w}\leftarrow p_{b_{i}}^{w}+R_{b_{i}}^{w}\delta p_{b_{i}} & p_{c}^{b}\leftarrow p_{c}^{b}+R_{c}^{b}\delta p_{c}^{b},\\ v_{b_{i}}^{w}\leftarrow v_{b_{i}}^{w}+\delta v_{b_{i}},\;\;\;\;\; & p_{k}^{w}\;\;\leftarrow p_{k}^{w}+\delta p_{k}^{w},\;\;\;\\ \delta b_{g_{i}}\leftarrow \delta b_{g_{i}}+\tilde{\delta }b_{g_{i}},\;\;\;\;\; & t_{d}\leftarrow t_{d}+\delta t_{d},\;\;\;\;\;\\ \delta b_{a_{i}}\leftarrow \delta b_{a_{i}}+\tilde{\delta }b_{a_{i}}.\;\;\;\;\; & \; \end{array}$$

Based on these update equations, we derive the Jacobians w.r.t. the vectors $\delta \phi_{b_{i}}$, $\delta p_{b_{i}}$, $\delta v_{b_{i}}$, $\tilde{\delta }b_{g_{i}}$, $\tilde{\delta }b_{a_{i}}$, $\delta \phi_{c}^{b}$, $\delta p_{c}^{b}$, $\delta p_{k}^{w}$, and $\delta t_{d}$.

### Appendix A.4. Jacobians of Rotation Errors

The Jacobians of rotation error $e_{rot}$ between consecutive keyframes *i* and *j* w.r.t. the vectors $\delta \phi_{c}^{b}$, $\tilde{\delta }b_{g_{i}}$, and $\delta t_{d}$ are as follows.

By letting

$\phi_{1}^{\prime }=\mathrm{Log}\left({\left(\Delta {\bar{R}}_{ij}\mathrm{Exp}\left(J_{\Delta {\bar{R}}_{ij}}^{g}\delta b_{g}\right)\right)}^{T}\right)$,

$\phi_{2}^{\prime }=\mathrm{Log}\left(R_{2}^{\prime }\right)=\mathrm{Log}\left(\mathrm{Exp}\left(-\omega_{c_{i}}t_{d}\right)R_{w}^{c_{i}}R_{c_{j}}^{w}\mathrm{Exp}\left(\omega_{c_{j}}t_{d}\right)\right)$,

$\phi_{3}^{\prime }=\mathrm{Log}\left(\Delta {\bar{R}}_{ij}^{T}R_{c}^{b}R_{2}^{\prime }R_{b}^{c}\right)$, we have:

(A10) $$\begin{array}{cc} & e_{rot}\left(R_{c}^{b}\mathrm{Exp}\left(\delta \phi_{c}^{b}\right)\right)\\ & \;\;\overset{\left(A3\right)}{=}\mathrm{Log}\left(\mathrm{Exp}\left(\phi_{1}^{\prime }\right)\mathrm{Exp}\left(R_{c}^{b}\mathrm{Exp}\left(\delta \phi_{c}^{b}\right)\phi_{2}^{\prime }\right)\right)\\ & \;\;\overset{\left(A2\right)}{\approx }\mathrm{Log}\left(\mathrm{Exp}\left(\phi_{1}^{\prime }\right)\mathrm{Exp}\left(R_{c}^{b}\phi_{2}^{\prime }+R_{c}^{b}{\delta \phi_{c}^{b}}^{\wedge }\phi_{2}^{\prime }\right)\right)\\ & \;\;\overset{\left(A5\right)}{\approx }\mathrm{Log}\left(\mathrm{Exp}\left(\phi_{1}^{\prime }\right)\mathrm{Exp}\left(R_{c}^{b}\phi_{2}^{\prime }\right)\mathrm{Exp}\left(J_{r}\left(R_{c}^{b}\phi_{2}^{\prime }\right)R_{c}^{b}{\delta \phi_{c}^{b}}^{\wedge }\phi_{2}^{\prime }\right)\right)\\ & =\;\mathrm{Log}\left(\mathrm{Exp}\left(e_{rot}\left(R_{c}^{b}\right)\right)\mathrm{Exp}\left(-J_{r}\left(R_{c}^{b}\phi_{2}^{\prime }\right)R_{c}^{b}{\phi_{2}^{\prime }}^{\wedge }\delta \phi_{c}^{b}\right)\right)\\ & \;\overset{\left(A4\right)}{\approx }e_{rot}\left(R_{c}^{b}\right)-J_{r}^{-1}\left(e_{rot}\left(R_{c}^{b}\right)\right)J_{r}\left(R_{c}^{b}\phi_{2}^{\prime }\right)R_{c}^{b}{\phi_{2}^{\prime }}^{\wedge }\delta \phi_{c}^{b}, \end{array}$$

(A11) $$\begin{array}{cc} & e_{rot}\left(\delta b_{g_{i}}+\tilde{\delta }b_{g_{i}}\right)\\ & =\mathrm{Log}\;\left(\mathrm{Exp}\;\left(\;-J_{\Delta {\bar{R}}_{ij}}^{g}\;\cdot \;\left(\delta b_{g_{i}}+\tilde{\delta }b_{g_{i}}\right)\right)\mathrm{Exp}\left(\phi_{3}^{\prime }\right)\right)\\ & \;\;\overset{\left(A5\right)}{\approx }\;\;\mathrm{Log}\;\left(\mathrm{Exp}\;\left(\;-J_{l}^{b_{g}}J_{\Delta {\bar{R}}_{ij}}^{g}\tilde{\delta }b_{g_{i}}\;\right)\;\mathrm{Exp}\;\left(\;-J_{\Delta {\bar{R}}_{ij}}^{g}\delta b_{g_{i}}\;\right)\;\mathrm{Exp}\left(\phi_{3}^{\prime }\right)\right)\\ & =\mathrm{Log}\;\left(\mathrm{Exp}\;\left(\;-J_{l}^{b_{g}}J_{\Delta {\bar{R}}_{ij}}^{g}\tilde{\delta }b_{g_{i}}\;\right)\mathrm{Exp}\left(e_{rot}\left(\delta b_{g_{i}}\right)\right)\right)\\ & \;\overset{\left(A4\right)}{\approx }e_{rot}\left(\delta b_{g_{i}}\right)-J_{l}^{-1}\left(e_{rot}\left(\delta b_{g_{i}}\right)\right)J_{l}^{b_{g}}J_{\Delta {\bar{R}}_{ij}}^{g}\tilde{\delta }b_{g_{i}}. \end{array}$$

Here, the shorthand $J_{l}^{b_{g}}≐J_{l}\left(-J_{\Delta {\bar{R}}_{ij}}^{g}\delta b_{g_{i}}\right)$ is used in the deduction.

By letting $R_{1}^{\prime \prime }={\left(\Delta {\bar{R}}_{ij}\mathrm{Exp}\left(J_{\Delta {\bar{R}}_{ij}}^{g}\delta b_{g}\right)\right)}^{T}\;R_{c}^{b}$, $R_{2}^{\prime \prime }=R_{w}^{c_{i}}R_{c_{j}}^{w}$, and $R_{3}^{\prime \prime }=R_{b}^{c}$, we have:

(A12) $$\begin{array}{cc} & e_{rot}\left(t_{d}+\delta t_{d}\right)\\ & =\mathrm{Log}\left(R_{1}^{\prime \prime }\mathrm{Exp}\left(-\omega_{c_{i}}\left(t_{d}\;+\delta t_{d}\right)\right)R_{2}^{\prime \prime }\mathrm{Exp}\left(\omega_{c_{j}}\left(t_{d}\;+\delta t_{d}\right)\right)R_{3}^{\prime \prime }\right)\\ & \;\;\overset{\left(A5\right)}{\approx }\mathrm{Log}(R_{1}^{\prime \prime }\mathrm{Exp}\left(-J_{l}^{i}\omega_{c_{i}}\delta t_{d}\right)\mathrm{Exp}\left(-\omega_{c_{i}}t_{d}\right)R_{2}^{\prime \prime }\\ & \;\;\;\;\cdot \mathrm{Exp}\left(\omega_{c_{j}}t_{d}\right)\mathrm{Exp}\left(J_{r}^{j}\omega_{c_{j}}\delta t_{d}\right)R_{3}^{\prime \prime })\\ & \;\;\overset{\left(A3\right)}{=}\mathrm{Log}(\mathrm{Exp}\left(-R_{1}^{\prime \prime }J_{l}^{i}\omega_{c_{i}}\delta t_{d}\right)R_{1}^{\prime \prime }\mathrm{Exp}\left(-\omega_{c_{i}}t_{d}\right)R_{2}^{\prime \prime }\\ & \;\;\;\;\cdot \mathrm{Exp}\left(\omega_{c_{j}}t_{d}\right)R_{3}^{\prime \prime }\mathrm{Exp}\left({R_{3}^{\prime \prime }}^{T}J_{r}^{j}\omega_{c_{j}}\delta t_{d}\right))\\ & =\mathrm{Log}(\mathrm{Exp}\left(-R_{1}^{\prime \prime }J_{l}^{i}\omega_{c_{i}}\delta t_{d}\right)\mathrm{Exp}\left(e_{rot}\left(t_{d}\right)\right)\\ & \;\;\;\cdot \mathrm{Exp}({R_{3}^{\prime \prime }}^{T}J_{r}^{j}\omega_{c_{j}}\delta t_{d}))\\ & \;\;\overset{\left(A3\right)}{=}\mathrm{Log}(\mathrm{Exp}\left(e_{rot}\left(t_{d}\right)\right)\mathrm{Exp}\left(-{\mathrm{Exp}(e_{rot}\left(t_{d}\right))}^{T}R_{1}^{\prime \prime }J_{l}^{i}\omega_{c_{i}}\delta t_{d}\right)\\ & \;\;\;\cdot \mathrm{Exp}({R_{3}^{\prime \prime }}^{T}J_{r}^{j}\omega_{c_{j}}\delta t_{d}))\\ & =\mathrm{Log}\left(\mathrm{Exp}\left(e_{rot}\left(t_{d}\right)\right)\mathrm{Exp}\left(D\;\cdot \;\delta t_{d}\right)\mathrm{Exp}\left(E\;\cdot \;\delta t_{d}\right)\right)\\ & \;\;\overset{\left(A2\right)}{\approx }\mathrm{Log}\left(\mathrm{Exp}\left(e_{rot}\left(t_{d}\right)\right)\left(I+{\left(D+E\right)}^{\wedge }\delta t_{d}\right)\right)\\ & \;\;\overset{\left(A2\right)}{\approx }\mathrm{Log}\left(\mathrm{Exp}\left(e_{rot}\left(t_{d}\right)\right)\mathrm{Exp}\left(\left(D+E\right)\delta t_{d}\right)\right)\\ & \;\;\overset{\left(A4\right)}{\approx }e_{rot}\left(t_{d}\right)+J_{r}^{-1}\left(e_{rot}\left(t_{d}\right)\right)\left(D+E\right)\delta t_{d}. \end{array}$$

Here, $J_{l}^{i}≐J_{l}\left(-\omega_{c_{i}}t_{d}\right)$, $J_{r}^{j}≐J_{r}\left(\omega_{c_{j}}t_{d}\right)$, $D≐-{\mathrm{Exp}(e_{rot}\left(t_{d}\right))}^{T}R_{1}^{\prime \prime }J_{l}^{i}\omega_{c_{i}}$, and $E≐{R_{3}^{\prime \prime }}^{T}J_{r}^{j}\omega_{c_{j}}$ are used in the deduction. In summary, the Jacobians of $e_{rot}$ are:

$$\begin{array}{ccc} \frac{\partial e_{rot}}{\partial \delta \phi_{c}^{b}} & =-J_{r}^{-1}\left(e_{rot}\left(R_{c}^{b}\right)\right)J_{r}\left(R_{c}^{b}\phi_{2}^{\prime }\right)R_{c}^{b}{\phi_{2}^{\prime }}^{\wedge } & \;\\ \frac{\partial e_{rot}}{\partial \tilde{\delta }b_{g_{i}}} & =-J_{l}^{-1}\left(e_{rot}\left(\delta b_{g_{i}}\right)\right)J_{l}^{b_{g}}J_{\Delta {\bar{R}}_{ij}}^{g}, & \;\\ \frac{\partial e_{rot}}{\partial \delta t_{d}} & =J_{r}^{-1}\left(e_{rot}\left(t_{d}\right)\right)\left(D+E\right). & \; \end{array}$$

### Appendix A.5. Jacobians of Feature Residual Errors

We define the residual error between the reprojection of $p_{k}^{w}$ and the pixel location $u_{k}^{i}$ of the matched feature as $r_{C_{ik}}=u_{k}^{i}-\pi \left(p_{k}^{c_{i}}\right)$. Here, $p_{k}^{c_{i}}$ is the *k*th point observed at the *i*th keyframe. It is expressed in the local camera frame. Since $b_{a_{i}}$ does not appear in $r_{C_{ik}}$, the Jacobian of $r_{C_{ik}}$ w.r.t. $\tilde{\delta }b_{a_{i}}$ is zero. By representing $p_{k}^{c_{i}}$ using ${\left[X\;Y\;Z\right]}^{T}$, the Jacobians of $r_{C_{ik}}$ w.r.t. the system states are:

(A13) $$\frac{\partial r_{C_{ik}}}{\partial (\cdot )}=\frac{\partial r_{C_{ik}}}{\partial p_{k}^{c_{i}}}\frac{\partial p_{k}^{c_{i}}}{\partial (\cdot )}=\;-\frac{1}{Z}\;\left[\begin{array}{ccc} f_{x} & 0 & -f_{x}\;\cdot \;X/Z\\ 0 & f_{y} & -f_{y}\;\cdot \;Y/Z \end{array}\right]\;\cdot \frac{\partial p_{k}^{c_{i}}}{\partial (\cdot )},$$

where $f_{x}$ and $f_{y}$ are the focal lengths of the camera. $\partial r_{C_{ik}}/\partial p_{k}^{c_{i}}$ is derived from the projection function of a pinhole camera model.

Since $p_{k}^{c_{i}}$ is linear in $v_{b_{i}}^{w}$ and $p_{k}^{w}$ and the retraction is simply a vector sum, the Jacobians of $p_{k}^{c_{i}}$ w.r.t. $\delta v_{b_{i}}^{w}$, $\delta p_{k}^{w}$ are simplified as the matrix coefficients of $v_{b_{i}}^{w}$ and $p_{k}^{w}$. We only need to focus on the following remaining Jacobians:

(A14) $$\begin{array}{cc} & p_{k}^{c_{i}}\left(p_{b_{i}}^{w}+R_{b_{i}}^{w}\delta p_{b_{i}}\right)\\ & =R_{b}^{c}\mathrm{Exp}\left({\tilde{\omega }}_{b_{i}}t_{d}\right){R_{b_{i}}^{w}}^{T}\left(C-R_{b_{i}}^{w}\delta p_{b_{i}}\right)+p_{b}^{c}\\ & =p_{k}^{c_{i}}\left(p_{b_{i}}^{w}\right)-R_{b}^{c}\mathrm{Exp}\left({\tilde{\omega }}_{b_{i}}t_{d}\right)\delta p_{b_{i}}, \end{array}$$

(A15) $$\begin{array}{cc} & p_{k}^{c_{i}}\left(R_{b_{i}}^{w}\mathrm{Exp}\left(\delta \phi_{b_{i}}\right)\right)\\ & =R_{b}^{c}\mathrm{Exp}\left({\tilde{\omega }}_{b_{i}}t_{d}\right)\mathrm{Exp}\left(-\delta \phi_{b_{i}}\right){R_{b_{i}}^{w}}^{T}C+p_{b}^{c}\\ & \;\;\overset{\left(A2\right)}{\approx }R_{b}^{c}\mathrm{Exp}\left({\tilde{\omega }}_{b_{i}}t_{d}\right)\left(I-{\delta \phi_{b_{i}}}^{\wedge }\right){R_{b_{i}}^{w}}^{T}C+p_{b}^{c}\\ & =p_{k}^{c_{i}}\left(R_{b_{i}}^{w}\right)+R_{b}^{c}\mathrm{Exp}\left({\tilde{\omega }}_{b_{i}}t_{d}\right){\left({R_{b_{i}}^{w}}^{T}C\right)}^{\wedge }\delta \phi_{b_{i}}, \end{array}$$

(A16) $$\begin{array}{cc} & p_{k}^{c_{i}}\left(p_{c}^{b}+R_{c}^{b}\delta p_{c}^{b}\right)\\ & =R_{b}^{c}\mathrm{Exp}\left({\tilde{\omega }}_{b_{i}}t_{d}\right){R_{b_{i}}^{w}}^{T}C-{R_{c}^{b}}^{T}\left(p_{c}^{b}+R_{c}^{b}\delta p_{c}^{b}\right)\\ & =p_{k}^{c_{i}}\left(p_{c}^{b}\right)-\delta p_{c}^{b}, \end{array}$$

(A17) $$\begin{array}{cc} & p_{k}^{c_{i}}\left(R_{c}^{b}\mathrm{Exp}\left(\delta \phi_{c}^{b}\right)\right)\\ & =\mathrm{Exp}\left(-\delta \phi_{c}^{b}\right)R_{b}^{c}\mathrm{Exp}\left({\tilde{\omega }}_{b_{i}}t_{d}\right){R_{b_{i}}^{w}}^{T}C+\mathrm{Exp}\left(-\delta \phi_{c}^{b}\right)p_{b}^{c}\\ & \;\;\overset{\left(A2\right)}{\approx }\left(I-{\left(\delta \phi_{c}^{b}\right)}^{\wedge }\right)p_{k}^{c_{i}}\left(R_{c}^{b}\right)\\ & =p_{k}^{c_{i}}\left(R_{c}^{b}\right)+{\left(p_{k}^{c_{i}}\left(R_{c}^{b}\right)\right)}^{\wedge }\delta \phi_{c}^{b}, \end{array}$$

(A18) $$\begin{array}{cc} & p_{k}^{c_{i}}\left(t_{d}+\delta t_{d}\right)\\ & =R_{b}^{c}\mathrm{Exp}\left({\tilde{\omega }}_{b_{i}}t_{d}+{\tilde{\omega }}_{b_{i}}\delta t_{d}\right){R_{b_{i}}^{w}}^{T}\;\;\left(C+v_{b_{i}}^{w}\delta t_{d}\right)+p_{b}^{c}\\ & \;\;\overset{\left(A5\right)}{\underset{\left(A2\right)}{\approx }}R_{b}^{c}\mathrm{Exp}\left({\tilde{\omega }}_{b_{i}}t_{d}\right)\left(I+{\left(J_{r}\left({\tilde{\omega }}_{b_{i}}t_{d}\right){\tilde{\omega }}_{b_{i}}\delta t_{d}\right)}^{\wedge }\right){R_{b_{i}}^{w}}^{T}\\ & \;\;\;\cdot \;\left(C+v_{b_{i}}^{w}\delta t_{d}\right)+p_{b}^{c}\\ & \approx p_{k}^{c_{i}}\left(t_{d}\right)+R_{b}^{c}\mathrm{Exp}\left({\tilde{\omega }}_{b_{i}}t_{d}\right)\\ & \;\;\;\cdot \;\left({R_{b_{i}}^{w}}^{T}v_{b_{i}}^{w}+{\left(J_{r}\left({\tilde{\omega }}_{b_{i}}t_{d}\right)\;\cdot \;{\tilde{\omega }}_{b_{i}}\right)}^{\wedge }{R_{b_{i}}^{w}}^{T}C\right)\delta t_{d}, \end{array}$$

(A19) $$\begin{array}{cc} & p_{k}^{c_{i}}\left(\delta b_{g_{i}}+\tilde{\delta }b_{g_{i}}\right)\\ & =R_{b}^{c}\mathrm{Exp}\left({\tilde{\omega }}_{b_{i}}t_{d}-\tilde{\delta }b_{g_{i}}t_{d}\right){R_{b_{i}}^{w}}^{T}C+p_{b}^{c}\\ & \;\;\overset{\left(A5\right)}{\underset{\left(A2\right)}{\approx }}R_{b}^{c}\mathrm{Exp}\left({\tilde{\omega }}_{b_{i}}t_{d}\right)\left(I-{\left(J_{r}\left({\tilde{\omega }}_{b_{i}}t_{d}\right)\tilde{\delta }b_{g_{i}}t_{d}\right)}^{\wedge }\right){R_{b_{i}}^{w}}^{T}C+p_{b}^{c}\\ & =p_{k}^{c_{i}}\left(\delta b_{g_{i}}\right)+R_{b}^{c}\mathrm{Exp}\left({\tilde{\omega }}_{b_{i}}t_{d}\right){\left({R_{b_{i}}^{w}}^{T}C\right)}^{\wedge }J_{r}\left({\tilde{\omega }}_{b_{i}}t_{d}\right)\tilde{\delta }b_{g_{i}}t_{d}. \end{array}$$

Here, we used the shorthand $C≐p_{k}^{w}\;-p_{b_{i}}^{w}+v_{b_{i}}^{w}t_{d}$ for convenience. $J_{r}\left({\tilde{\omega }}_{b_{i}}t_{d}\right)$ is the right-Jacobian matrix of ${\tilde{\omega }}_{b_{i}}t_{d}$. In summary, the Jacobians of $p_{k}^{c_{i}}$ are:

$$\begin{array}{cc} \frac{\partial p_{k}^{c_{i}}}{\partial \delta p_{b_{i}}} & =-R_{b}^{c}\mathrm{Exp}\left({\tilde{\omega }}_{b_{i}}t_{d}\right),\;\;\;\;\;\;\;\;\;\;\;\;\frac{\partial p_{k}^{c_{i}}}{\partial \tilde{\delta }b_{a_{i}}}=0_{3\times 3},\\ \frac{\partial p_{k}^{c_{i}}}{\partial \delta v_{b_{i}}} & =R_{b}^{c}\mathrm{Exp}\left({\tilde{\omega }}_{b_{i}}t_{d}\right){R_{b_{i}}^{w}}^{T}t_{d},\;\;\;\;\;\;\;\frac{\partial p_{k}^{c_{i}}}{\partial \delta \phi_{c}^{b}}={\left(p_{k}^{c_{i}}\left(R_{c}^{b}\right)\right)}^{\wedge },\\ \frac{\partial p_{k}^{c_{i}}}{\partial \delta \phi_{b_{i}}} & =R_{b}^{c}\mathrm{Exp}\left({\tilde{\omega }}_{b_{i}}t_{d}\right){\left({R_{b_{i}}^{w}}^{T}C\right)}^{\wedge },\;\;\frac{\partial p_{k}^{c_{i}}}{\partial \delta p_{c}^{b}}=-I_{3\times 3},\\ \frac{\partial p_{k}^{c_{i}}}{\partial \delta p_{k}^{w}} & =R_{b}^{c}\mathrm{Exp}\left({\tilde{\omega }}_{b_{i}}t_{d}\right){R_{b_{i}}^{w}}^{T},\\ \frac{\partial p_{k}^{c_{i}}}{\partial \tilde{\delta }b_{g_{i}}} & =R_{b}^{c}\mathrm{Exp}\left({\tilde{\omega }}_{b_{i}}t_{d}\right)\;{\left(\;{R_{b_{i}}^{w}}^{T}C\right)}^{\wedge }J_{r}\left({\tilde{\omega }}_{b_{i}}t_{d}\right)t_{d},\\ \frac{\partial p_{k}^{c_{i}}}{\partial \delta t_{d}} & =R_{b}^{c}\mathrm{Exp}\left({\tilde{\omega }}_{b_{i}}t_{d}\right)\;\left(\;{R_{b_{i}}^{w}}^{T}\;v_{b_{i}}^{w}\;+\;{\left(J_{r}\left({\tilde{\omega }}_{b_{i}}t_{d}\right)\;\cdot \;{\tilde{\omega }}_{b_{i}}\;\right)}^{\wedge }\;{R_{b_{i}}^{w}}^{T}\;C\;\right)\;. \end{array}$$
